# Role of Actin Filaments in Correlating Nuclear Shape and Cell Spreading

**DOI:** 10.1371/journal.pone.0107895

**Published:** 2014-09-24

**Authors:** Renu Vishavkarma, Swetavalli Raghavan, Chandrashekar Kuyyamudi, Abhijit Majumder, Jyotsna Dhawan, Pramod A. Pullarkat

**Affiliations:** 1 Soft Condensed Matter group, Raman Research Institute, Bangalore, Karnataka, India; 2 Institute for Stem Cell Biology and Regenerative Medicine, National Centre for Biological Sciences (TIFR), Bangalore, Karnataka, India; Semmelweis University, Hungary

## Abstract

It is well known that substrate properties like stiffness and adhesivity influence stem cell morphology and differentiation. Recent experiments show that cell morphology influences nuclear geometry and hence gene expression profile. The mechanism by which surface properties regulate cell and nuclear properties is only beginning to be understood. Direct transmission of forces as well as chemical signalling are involved in this process. Here, we investigate the formal aspect by studying the correlation between cell spreading and nuclear deformation using Mesenchymal stem cells under a wide variety of conditions. It is observed that a robust quantitative relation holds between the cell and nuclear projected areas, irrespective of how the cell area is modified or when various cytoskeletal or nuclear components are perturbed. By studying the role of actin stress fibers in compressing the nucleus we propose that nuclear compression by stress fibers can lead to enhanced cell spreading due to an interplay between elastic and adhesion factors. The significance of myosin-II in regulating this process is also explored. We demonstrate this effect using a simple technique to apply external compressive loads on the nucleus.

## Introduction

It is now a well-established fact that cellular morphology, function and organization can be influenced at a fundamental level by substrate properties like adhesion and elasticity [1]–[4]. Neuronal cells, for example, show a preference for soft substrates with moduli close to that of the brain, whereas fibroblasts show an affinity for stiffer substrates [5]. In recent years, it has been shown that substrate properties influence lineage specification in stem cells [6]–[8]. Soft substrates seem to favor differentiation into neuronal cells whereas stiff substrates generate osteoblasts [6]. Further, It has also been observed that conditions of cell spreading alone may influence the process of lineage determination [9] and cell spreading is influenced by substrate elasticity [1], [6]. Remarkably, it has also been shown that direct application of mechanical stresses to the cell nucleus may influence gene expression [10] and nuclear architecture may be regulated by cytoskeletal stresses [11]–[14].

In adherent cells, nuclear deformations are coupled to the cell cytoskeleton, especially via actin stress fibers [12], [15], [16]. The mechanisms by which nuclear deformations are regulated in a substrate dependent manner, and the exact role of cytoskeleton in this process is only beginning to be understood. There are two possible mechanisms to explain the coupling between the cell and the nuclear geometry via cytoskeleton: (a) compressive loading due to stress fibers running over the nucleus [12], and (b) lateral pulling by the direct coupling between adhesion proteins and nuclear membrane via cytoskeletal components [17]. Experiments where cells are grown on adhesive islands of different shapes or adhesive strips show that variation in cell spreading is transmitted to the nucleus by actin stress fibers and results in nuclear deformation [11], [12], [14]. It has been demonstrated that when cells are spread on highly anisotropic patches, the nucleus is elongated along the long axis of the pattern and actin stress fibers running on either sides of the nucleus are responsible for the observed deformation [14]. Stress fibers have also been observed to run over the nucleus, and ablation of these fibers result in reorganization of nuclear structures [12], [16]. All these results point towards a mechanical connection between the actin cytoskeleton and nucleus, which could regulate nuclear deformations. Myosin II seems to be crucially involved in this process as cell differentiation is hindered by its inhibition using Blebbistatin [6].

Further, it is known that cell spreading and nuclear geometry are related and change in a correlated manner when growth conditions are changed or cells are detached using trypsin [17], [18]. But the mechanism which links nuclear deformation to cell spreading is not understood.

In this article we explore the mechanism that links cell spreading to nuclear deformation. Our aim is to understand how actin cytoskeleton regulates both nuclear geometry and cell spreading in a tightly coupled manner. For this, we first quantify the cell spreading area and nuclear projected area of Mouse Mesenchymal stem cells (mMSCs) under different spreading conditions–cells grown on gels of different stiffnesses, during dynamics cell spreading, during trypsin mediated de-adhesion etc., and show that the two areas remain coupled. We then ask if cytoskeletal perturbations or nuclear perturbations can upset this coupling. Remarkably, we find that the cell area Vs. nuclear area data from all these experiments fall reasonably well on a single Master Curve without any scaling. By studying the response of these cells to an external compressive loading and with the aid of a simple theoretical model we conclude that compression of the nucleus by perinuclear stress fibers can aid cell spreading. We propose that this can arise due to an interplay between elastic stresses in the nucleus, tension generated by stress fibers or cortex and cell-substrate adhesion.

## Materials and Methods

### Ethics Statement

Mouse Mesenchymal stem cells (mMSCs) used in the experiments were prepared in Tulane University. These cells were obtained under a protocol approved by the Tulane University Institutional Animal Care and use Committee.

### Substrate preparation

Polyacrylamide (PAA) gels of about 300 *µ*m thickness were prepared on 18 mm activated circular coverslips. For activation, cleaned coverslips were kept in a solution consisting of 90% (v/v) absolute ethanol, 8% deionized Millipore water and 2% 3-Aminopropyltrimethoxysilane (Sigma) for 30 to 45 min., rinsed with Millipore water, incubated at 120°C for 1 hr., and finally treated with 0.5% Gluteraldehyde solution for 30 min. After rinsing, the coverslips were used within 24 hrs. to prepare gels. Substrate rigidity was controlled by adjusting the percentage of Bis-acrylamide (Sigma) from 0.5% to 0.03% in a solution of 10% Acrylamide (Sigma) and cured at room temperature [19]. Solutions with different concentrations of Acrylamide and Bis-Acrylamide were prepared and 200 *µ*l of the solution was put on the activated coverslip. To flatten the liquid droplet and therefore achieve a flat and smooth top surface of the gel, a cleaned untreated coverslip was put on the top of the liquid. Once the gel is polymerized, the top coverslip was removed with the help of fine tweezers and the substrates were flooded with Millipore water to avoid dehydration. To make the substrates compatible for cell adhesion, they were rinsed thoroughly with 50 mM HEPES buffer and then treated with a 10 mM solution of Sulfo-SANPAH (Pierce; Thermo Scientific) in HEPES for 15 min. under a 365 nm UV lamp (Pierce; Thermo scientific). A further 15 min. exposure to UV was done after rinsing with HEPES. The gels were then incubated overnight at 37°C with 50 *µ*g/ml fibronectin solution (Sigma).

Substrates were characterized using an Atomic force microscope (AFM) and the ball-indentation method, using Hertz equation to calculate the Young's modulus
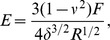
where 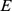 is the Young's modulus, 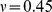 is the Poisson ratio [20], 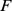 is the normal force, 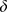 is the measured indentation of the ball, and 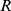 is the radius of the ball or the radius of curvature of the AFM tip. For the ball-indentation method, a solution of 2 *µ*m fluorescent beads in 1 ml HBSS was spread over the gel surface as a surface marker. Then a steel ball of radius 1.59 mm and weight 0.129 grams was placed on the surface as indenter. Indentations were measured using a calibrated microscope focus arrangement. Indentation values for the calibration using AFM were taken by applying a constant force on the gel surface using a spherical tip cantilever over a scanning area of 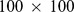 *µ*m^2^. The spring constant of the cantilever was 0.2 N/m and the diameter of the spherical tip was 1 *µ*m. This scanning was done to check if there are significant inhomogeneities, especially in weaker gels when probed at the scale of a focal adhesion. The rigidity values used in the plots are from the ball-indentation method. The calibration data are presented as Fig. S1 and Table S1.

### Cell culture

mMSCs were cultured in Alpha-MEM medium with 10% horse serum, 10% MSC certified FBS, 1% Glutamax and 1% PSG. For 3T3 fibroblast and C2C12 cells, DMEM with 10% and 20% FBS respectively was used. 1% PSG and 1% Glutamax was added to all growth media. All the reagents for cell culture are from Gibco Invitrogen. All cells were incubated at 37°C with 5% CO_2_. Cells (8-10th passage for mMSC) with 50–60% confluence were imaged after 12 hrs. of plating on gel surfaces.

### Drug treatment

#### Cytoskeleton perturbation

For myosin-II inhibition, cell suspensions were prepared in culture medium mixed with 20 *µ*M blebbistatin (Blebbi) (Sigma) and cultured on fibronectin coated gel surfaces with different stiffnesses. After being incubated for 8 hrs, cells were imaged for nucleus and cell spreading. To re-affirm these results, experiments were performed by adding blebbistatin to normal cells, already spread on the gel surface and the change in spreading was recorded. Microtubule disruption was performed using Nocodazole (Sigma) at 17 *µ*M conc., and actin disruptions were carried out using Latrunculin-A (Sigma) at concentrations of 80 *n*M and 0.67 *µ*M. Observations were made after 15 min. of incubation at 37°C. In all cases, stock solutions were prepared in DMSO with the final maximum DMSO concentration in culture less than 0.1%. At this concentration DMSO did not affect cell spreading (see Fig. S9).

#### siRNA Transfection

Lamin A/C silencing siRNA (Thermo Scientific, cat. no. D-001050-01-05) with transfection indicator was used to transfect mMSCs. Cells were plated at 50% confluence on fibronectin coated circular cover glass attached to punched petri-plates. 2.5 *µ*g of siRNA was diluted in 150 *µ*l of OptiMEM (solution A) and 12.5 *µ*l of Dharma FECT (ThermoScientific) was diluted in 150 *µ*l of OptiMEM (solution B). Solutions were incubated for 5 minutes at room temperature. Solution B was gently mixed into solution A (hereafter, referred to as transfection mixture) and incubated at room temperature for 30 minutes. Cells were washed with OptiMEM (Invitrogen) after removing the growth media. Transfection mixture was added drop wise to the cell plate and shaken gently. An additional 1.6 ml of OptiMEM medium is added and the cells were incubated at 37°C. Transfection medium was replaced with complete growth medium (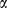-MEM; Invitrogen) after 5 hours. Transfected cells were imaged for nuclear and cell projected areas after 48 hrs of transfection.

#### Inhibition of HDAC activity

mMSC cells were treated with 6.6 *µ*M trichostatin A (TSA) in a humidified incubator maintained at 37°C and 5% CO2 levels for 2–3 hours. Cells were then washed and fixed with 4% paraformaldehyde (PFA) and immunostained for actin and nucleus as discussed below. Alternatively, time-lapse of cells, treated with TSA and stained for nucleus, were recorded.

### Trypsin de-adhesion

Cells were cultured for 24 hrs. on fibronectin coated coverslips. They were rinsed with HBSS w/o Ca^++^ and Mg^++^ and treated with Trypsin 1X (Gibco Invitrogen) solution. Cells were imaged immediately after the treatment.

### Immunostaining

Cells were fixed with 4% paraformaldehyde in phosphate buffered saline (Invitrogen) for 15 min. and permeabilized with 0.1% Trition X-100 (Thermo Scientific) for 10 min. at room temperature. 5% FBS (Gibco Invitrogen), and 5% Bovine Serum Albumin (Sigma Aldrich), in phosphate buffered saline was used to block non-specific binding for 1 hour. Primary antibody, anti-vinculin (Sigma Aldrich, cat. no. V9131) at 1∶500, and secondary antibody Alexa-Fluor goat-anti-mouse 488 (Molecular Probes-Invitrogen, cat. no. A1101) at 1∶800 was used to label the focal adhesions. Hoechst H33342 (Sigma Aldrich) and Phalloidin-tetramethylrhodamine (Fluka) were used for staining the nuclei and actin-filaments, respectively.

### Traction force microscopy

The following procedure was followed in order to obtain gels with a layer of fluorescent beads for performing traction force microscopy. Coverslips were cleaned and coated with 20 *µ*g/ml fibronectin solution for 30 min. at 37°C in a humidified chamber. This was done to make the coverslips adhesive so that the beads don't fly off while spin coating. After 30 min of incubation the solution was removed and 200 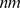 size fluorescent beads (Molecular Probes, cat. no. F8809) were spin coated on the coverslips at 1200 RPM for 3 min. The bead density was sufficient to have 15 beads in an area of 100 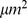. These coverslips were then used as a top plate to prepare PAA gel substrates as described earlier. An image of cells plated on such a surface is shown in Fig. S2. In order to estimate the traction under a focal adhesion, cell were transfected with Vinculin Venus (Addgene, cat. no. 27300) and grown on substrates with different rigidity. These cells were imaged using a normal fluorescence mode. Beads under a cell were imaged before and after trypsinization and the Matlab code described in the reference [21] was used to calculate the displacement field and the traction field under mature focal adhesions (FA) (Figs. S3 and S4). Focal complexes of nearly 20 cells were imaged for each gel rigidity (total 30–40 FAs). The average FA area for mature focal adhesions was found to be 4 *µ*m^2^ and forces due to these were calculated by multiplying the average traction with average area of FA.

### Imaging and Analysis

The cell and nuclear images were recorded in fluorescence using Calcein AM and Hoechst H33342 dyes respectively. Actin was imaged using Phalloidin-tetramethylrhodamine. Images for cell spreading and nuclear projected area were taken using an Olympus-IX71 microscope with a Andor Luca EM-CCD camera. Confocal images were recorded using a Ziess Meta 510 system. Images for dynamic cell spreading and trypsin de-adhesion were taken for live cells, whereas for confocal, cells were fixed with paraformaldehyde and permeabilized using 0.1% triton X-100. Image analysis to quantify cell spreading area and nuclear projected area was done using a custom written Matlab code. The code involved conversion of grayscale images into binary images by applying a threshold to the grayscale values and then finding area using an inbuilt Matlab function “regionprops”. Fiji-just ImageJ plugins were used for analyzing the phase contrast images from trypsin experiment and calculating the volume from confocal z-stacks. z-stacks were acquired using the confocal microscope with a resolution of 0.6 *µ*m (for thick cells) to 0.2 *µ*m (for thin cells) (about 50 slices per cell). The object counter plugin of Fiji-Just ImageJ [22] was used to calculate the nuclear volume. The plugin adds the voxels in each slice of the z-stack of an object to give volume.

## Results

### Relation between cell area and nucleus projected area

**Figure 1:**
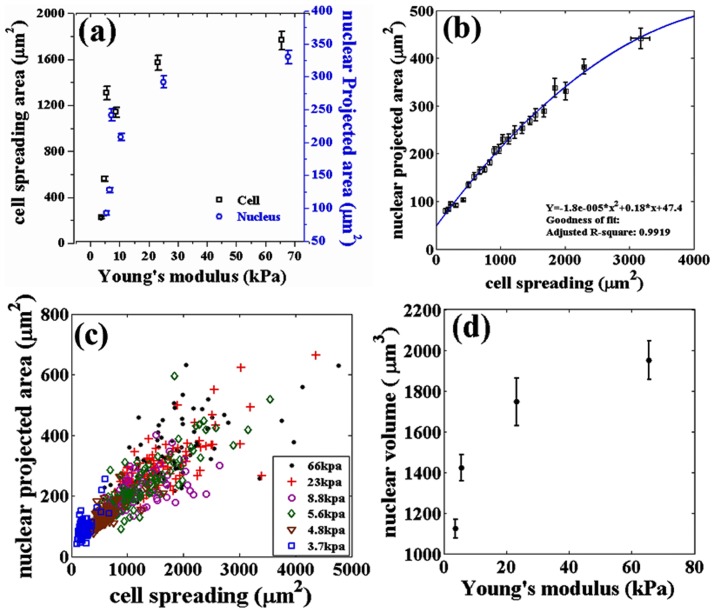
Variation of cell spreading, nuclear projected area and nuclear volume studied using gels of different stiffnesses. (a) Cell and nuclear projected area as a function of Young's modulus of the substrate. Each point is an average taken over 100 cells. (b) Dependence of nuclear projected area on cell spreading obtained after putting all the data from all rigidities together and then binning the data points for cell spreading. Note, the difference in maximum spread area between the two figures arises due to this pooling and binning of data according to cell spread area. Binning size used was 26 and 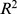 is calculated using the curve fitting toolbox, MATLAB. (c) Scatter plot (raw data) of the two areas of individual cells obtained from different substrates (same data as in a and b). Note that the range of measured cell area increases with substrate stiffness. (d) Nuclear volume as a function of the elastic modulus of the substrate measured from confocal stacks as describes in the text (20 cells for each data point). Error bars in all the plots represent mean 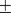 standard error (SE).

**Figure 2:**
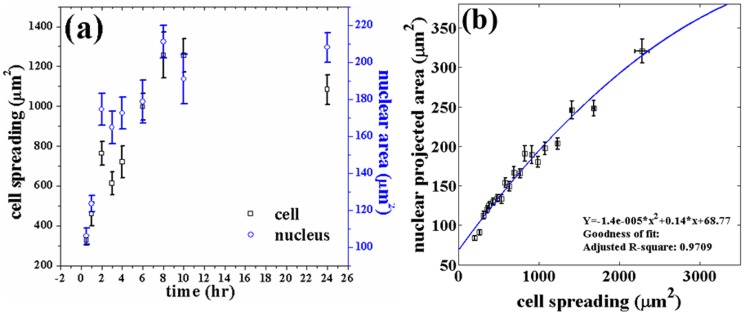
Variation of cell and nuclear projected areas studied as a function of dynamic cell spreading. (a) Cell area and nuclear projected area as a function of time for cells grown on plastic surfaces for up to 24 hrs. For each time point around 50–60 cells were imaged and the average values of cell spreading and nuclear projected area is plotted as a function of time. (b) Relation between the two areas obtained from the data shown in Fig. 2a. This plot is obtained by binning the cell areas of all 540 cells. Binning size used was 27 and 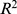 is calculated using the curve fitting toolbox, MATLAB. Note, the difference in maximum spread area between the two figures arises due to this pooling and binning of data according to cell spread area. The smooth line is the fit from Fig. 1 given for the sake of comparison. The error bars in (a) and (b) are mean 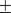 SE.

**Figure 3:**
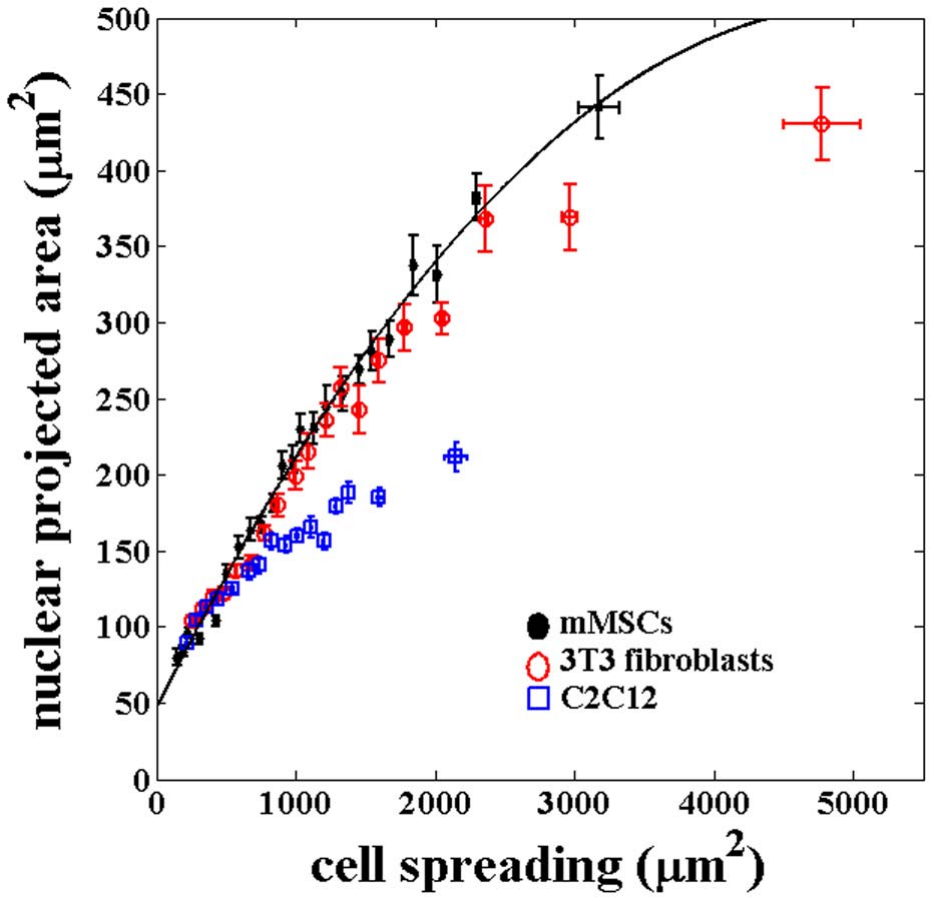
Plot of the cell and nuclear areas for three different cell types obtained using substrates with different stiffness. For each substrate stiffness, nearly 100 cells were imaged for each type of cell. The data from all the substrates for, each type of cell, is pooled together and plotted after binning as done previously for mMSCs. The error bars here represents 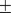 SE. The correlated behavior between cell and nuclear projected area seem to be roughly intact even across these different cell types although the nuclear projected areas is reaching saturation at different values.

We observed that mMSC plated on substrates with different stiffnesses exhibit increased cell spreading with increase in stiffness in the range of 3 to 70 kPa as shown in Fig. 1(a). The maximum stiffness sensitivity is in the 3 to 20 kPa range. This agrees with the observations in previous studies by various groups [2], [4], [6]. Nuclear projected area also follows a similar, highly correlated, behavior with stiffness (Fig. 1(a)). Figure 1(b) shows how the nuclear projected area increase with increase in cell spreading. This plot is obtained by binning all the data from different substrates according to their cell spread area. The raw data from individual substrates is shown in Fig. 1(c). In order to check the dependence of nuclear deformation (projected area) on cell spreading in a manner which is independent of substrate properties, including nature of adhesion, we plated mMSCs on cell culture treated petri dishes (Nunc) and recorded the cell and nuclear areas as a function of time as cell spreading progressed (Movie S1). Once again we obtained a nearly identical relation between the two recorded areas as shown in Fig. 2(a) and 2(b). In order to check if a similar relation would hold for other differentiated cell types, we performed experiments using 3T3 fibroblast and C2C12 cells cultured on substrates with different rigidities. We found that the correlated behavior of the two projected areas hold even for these cells but with different slopes as shown in Fig. 3 (Fig. S5).

#### Substrate stiffness dependence of nuclear volume

As can be seen from Fig. 1(d), the nuclear volume for cells grown on gels with different stiffnesses shows a trend very similar to the cell and nuclear projected area. These cells are grown on gels for more than 12 hrs. Similar type of increase in volume had also been observed for endothelial cells, when grown on patterned substrates with varying area and is attributed to enhanced DNA synthesis in well spread cells [11]. This increase in volume with stiffness could have important implications on nuclear compactness and hence in the regulation of gene expression.

When well spread cells on stiff substrates were detached using trypsin, the initially flattened nucleus becomes rounded within minutes, without any measurable change in its volume (Fig. S6). This suggests that at such short timescales the nucleus can deform by preserving its volume as growth effects do not come into play.

In order to further explore the relation between cell spreading, cytoskeletal organization and nuclear deformation we performed the following experiments.

### Trypsin de-adhesion experiments

**Figure 4:**
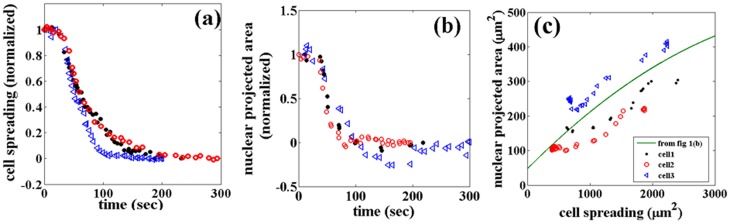
The relation between cell area and nuclear projected area during trypsin mediated deadhesion. (a, b) Changes in cell spreading and nuclear projected area (normalized values) as a function of time, obtained from individual cells (different symbols). Normalization is done using the formula 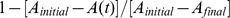. (c) Variation in nuclear projected area as a function of cell spreading for the same cells. The line is the same fit as in Fig. 1b, and is plotted for comparison. In some cases nuclear area shows an undershoot where the area decreases below the final value as seen in (b). Moreover, in some cases cell shrinkage precedes nuclear shrinkage as can be seen in (c).

To investigate the relation between cell and nuclear area in a manner which is independent of the substrate, we performed de-adhesion experiments using trypsin [23] and followed the nuclear deformation as a function of cell shrinkage (Movie S2). As seen in Fig. 4, the correlated variation in cell and nuclear projected areas remain similar to earlier cases during the entire de-adhesion process. These experiments reiterate the fact that there is a tight correlation between cell spreading and changes in the nucleus projected area (deformation) irrespective of the way the cell spreading is altered.

### Cytoskeleton and membrane perturbation experiments

**Figure 5:**
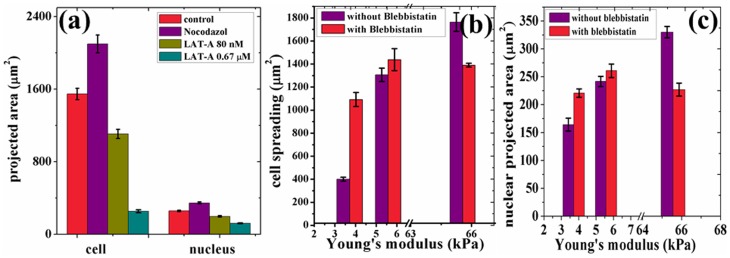
The robustness of the correlated behavior was tested against various cytoskeletal drug treatments. (a) Changes in cell and nuclear projected areas after microtubule disruption using Nocodazole and actin depolymerisation using Latrunculin-A. (b, c) Variation in the two areas after treatment with blebbistatin to deactivate myosin-II. Note that substrate sensitivity is significantly diminished after mysosin-II inhibition. Further, for all drug treatments, a change in cell area causes a correlated change in nuclear area. All the values for the cell area and nuclear projected area are taken from over 80–100 cells in each case and the error bars in the plots represent standard error. Student t-test values are 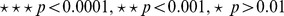 and the comparison is with control for (a) and with 65 kPa substrate for (b).

**Figure 6:**
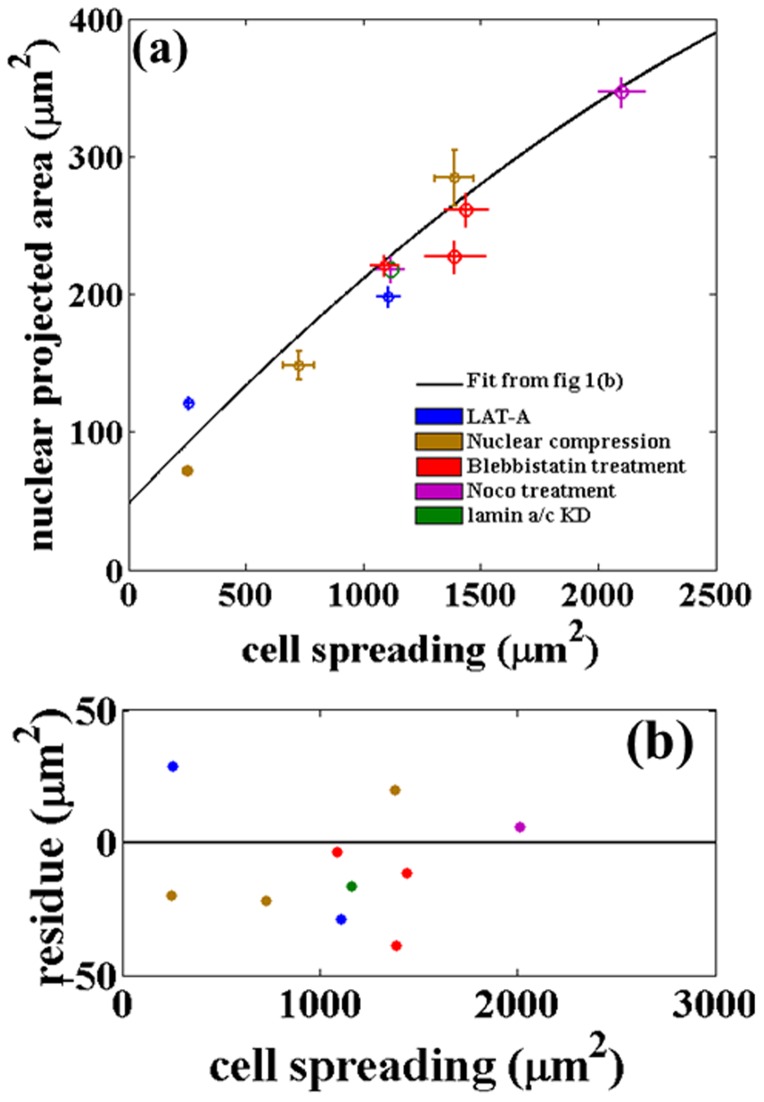
Changes in nuclear projected area plotted against cell spreading follow a Master Curve irrespective of the way cell spreading is altered or after perturbing different cytoskeletal components. (a) Nuclear projected area vs. cell spreading obtained after different perturbation experiments. The solid line is the fit obtained from Fig. 1b. (b) and 5(c). Data points from nuclear compression experiments show the change in the two areas with varying applied load as discussed in the text (30 cells were imaged outside the lens for zero load, 15 for 0.97 g lens, and 20 for 1.07 g lens). Error bars are mean 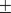 SE. (b) Residue plot for the data in (a). The residue plot shows the extend of deviations of the data obtained after perturbations from the fit obtained for the unperturbed cells. The agreement is remarkable considering the fact that perturbation experiments, although performed using specific drugs, lead to global reorganization of cellular components.

Can cytoskeletal perturbations de-couple the cell and nuclear shapes? To check this, we measured the correlation between cell spreading and nuclear projected area under different cytoskeletal perturbations and the results are summarized in Fig. 5 and Fig. 6. Microtubule disruption causes a slight increase in cell and nuclear projected areas, whereas actin depolymerization causes a concentration dependent reduction in these measured values (also see Fig. S7 for images and Movie S3). DMSO, used for mixing the chemicals, did not affect cell spreading (Fig. S9). All the experiments done for actin and microtubule perturbations were done on cells cultured on fibronectin coated coverslips. It is observed that these perturbations do not upset the correlation between the two areas significantly, even though cell spreading itself is affected. Myosin-II inhibition using blebbistatin were also performed using cells grown on substrates with different rigidities. The aim here was to study the influence of myosin-II on well spread and poorly spread cells. It was observed that non-muscle myosin-II inhibition make cells nearly insensitive towards the changes in elastic modulus of the substrate as can be seen in Figs. 5(b, c) (see Fig. S10 for images). In other words, cells on stiff substrates shrink in spreading, whereas those on soft substrates increase their spreading such that cells on every substrate show roughly the same spread area compared to control cells. Remarkably, all the changes in cell spreading caused by the disruption of cytoskeletal components or myosin inhibition were accompanied by changes in nuclear projected area consistent with the data obtained using different gels or by dynamic cell spreading as shown in the Master-Curve Fig. 6.

### Actin distribution and nuclear geometry

**Figure 7:**
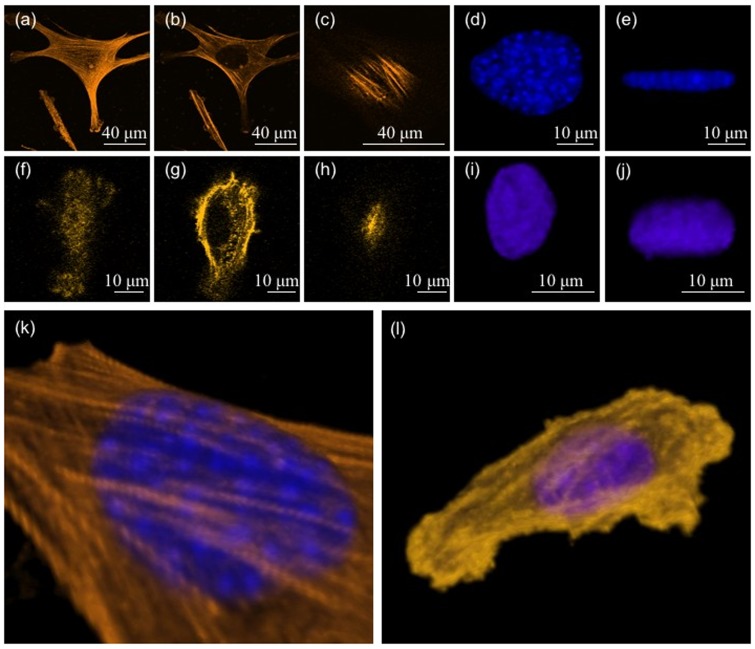
Confocal images showing the actin distribution and nuclear shape in cells spread on gels of two different stiffnesses. (a, b, c) Confocal images of a cell on a 70 kPa stiffness substrate. The images show actin filaments close to the plane of the substrate, in an approximate mid plane, and just above the nucleus respectively. (d, e) The nucleus of the same cell projected in the plane of the substrate and in a perpendicular plane respectively. (f–j) Similar observations of a cell spread on a soft substrate (3 kPa). Note the difference in the nuclear shape compared to the upper set. (k, l) 3D reconstruction of confocal images showing perinuclear stress fibers running over the nucleus in the case of the first cell (stiff substrate) and a predominantly cortical actin mesh in the case of the second cell (soft substrate). Images in k and l are 3D reconstructions of the cells shown in a–e and f–j respectively.

**Figure 8:**
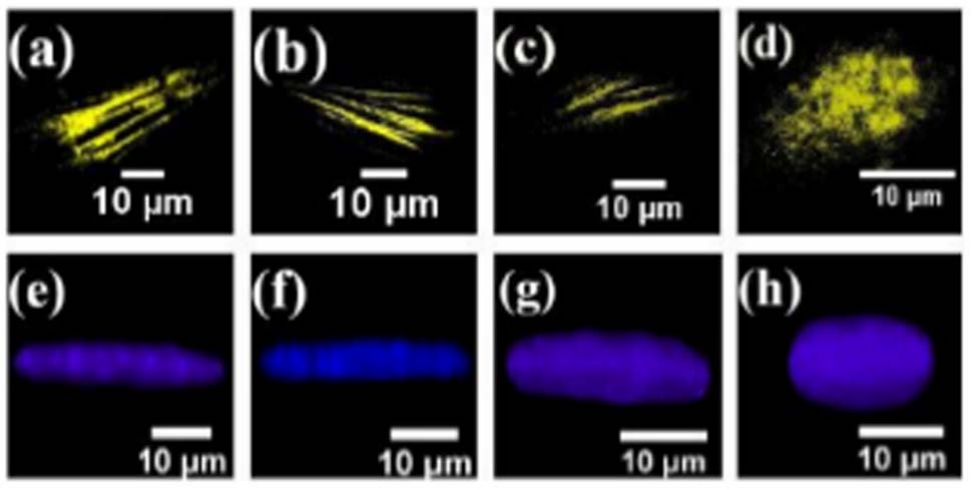
Changes in the perinuclear actin stress fibers and nuclear geometry for different rigidities. (a–d) The observed decrease in stress fiber density as function of substrate rigidity for substrates with elastic modulus 65 kPa, 23 kPa, 5 kPa and 3 kPa respectively (also see panel in Fig. S11). No stress fibers were observed in cell cultured on substrate with elastic modulus 3 kPa. (e–h) The transverse view (3D projections) of the nucleus under different compressive loading for the cells grown on substrates with different rigidities.

Detailed confocal imaging of cells spread on substrates with different stiffnesses reveal the following. On stiff substrates we observe two types of actin stress fibers. One set runs parallel to the substrate and lies very close to the plane of the substrate. These stress fibers lie below the nucleus (basal stress fibers). The other set ran over the nucleus (dorsal/perinuclear stress fibers) with the ends anchored at the substrate (Fig. 7(c) and 7(k)). Thus, the nucleus is tightly sandwiched between these two sets of stress fibers. The nucleus appears to be compressed between these two sets of stress fibers and is highly flattened on stiff substrates. As the stiffness is reduced, cell spreading, number of either type of stress fibers, and nuclear deformation reduced systematically as shown in Fig. 7 and Fig. 8 (also see images in Fig. S11). A precise quantification of the stress fiber density and thickness as a function of stiffness proved difficult as the distinction between stress fibers and background actin (cortex) became poor rapidly as the gel stiffness was reduced. As a rough estimate, on the hardest substrates, about 10
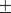
2 fibers could be seen running over the nucleus (Fig. S12). On the softest substrates (3 kPa) no stress fibers were visible and instead actin was distributed as an inhomogeneous cortical layer (Fig. 7(l)). The nucleus in this case was almost spherical Fig. 7(i) and 7(j). These observations suggest that the nuclear deformation seen on stiff substrates may be due to a compressive loading of the nucleus by perinuclear stress fibers as suggested in [12] for fibroblasts plated on adhesive strips. From this, we hypothesize that a reduction in stress fiber number and tension in these fibers may be responsible for the correlated reduction in nuclear compression and cell spreading as elaborated later.

### Nuclear perturbation experiments

**Figure 9:**
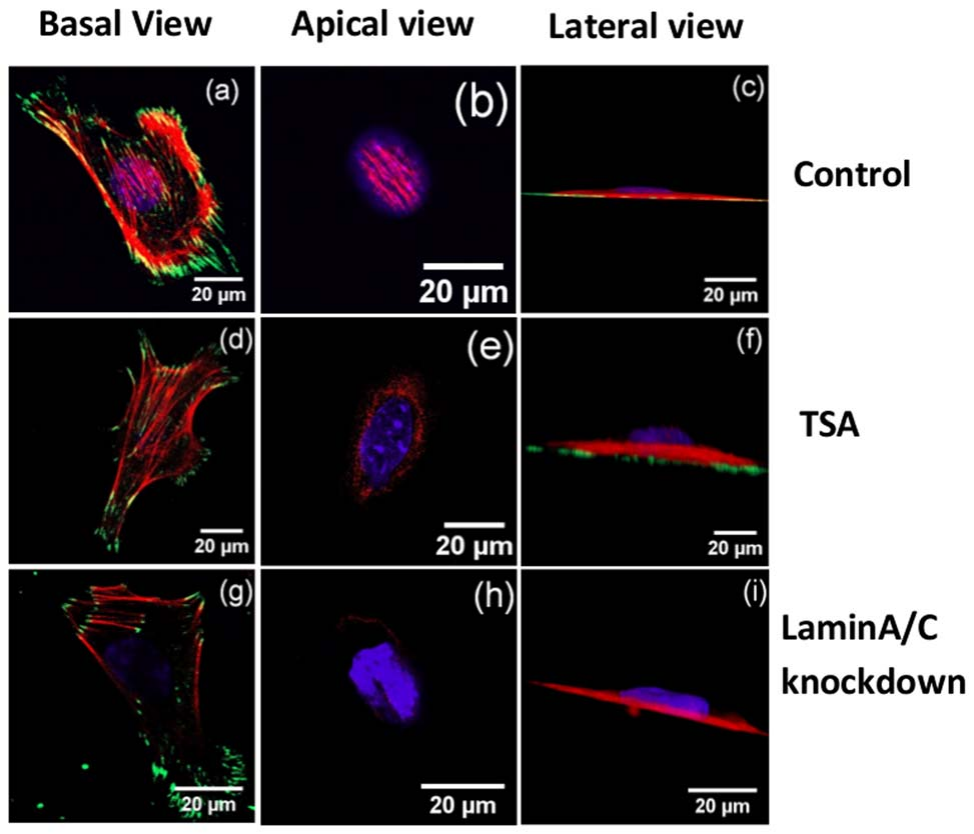
Confocal slices of cells after nuclear perturbations (control, TSA treatment and lamin a/c knock down). All the cells were cultured on fibronectin coated coverslips. Actin filaments, nucleus and focal adhesions are labelled in red, blue and green color respectively. It can be seen that perinuclear stress fibers are lost and the nucleus bulges out in treated cells.

Apart from cytoskeletal perturbations, we also performed nuclear perturbation experiments similar to that performed in [12] for fibroblasts. When nuclear lamin was disrupted by transfection with siRNA, major changes in actin organization and nuclear geometry were observed. After silencing lamin a/c no detectable perinuclear stress fibers were observed although most of the basal stress fibers appeared intact (Fig. 9(g), 9 (h)). This is because LINC complexes regulate perinuclear stress fibers and are lost with lamin a/c knockdown as shown in [12], [24]. Contrary to a flattened nuclei that is present on untreated cells (Fig. 9(c)), the nuclei of lamin a/c knockdown cells protruded outwards on the apical surface (Fig. 9(i)). Even after this perturbation, the cell and nuclear projected areas remain correlated as shown in Fig. 6. Experiments using lower (80 nM) concentrations of Latrunculin-A too disrupts the perinuclear actin preferentially (Fig. S8) and again a correlated reduction in cell and nuclear spread areas is observed as shown in the master curve Fig. 6 (Movie S3).

Experiments with TSA treatment to de-condense DNA revealed a significant reduction in the nuclear projected area accompanied by a corresponding decrease in cell area (Fig. S13). An increase in nuclear thickness and a prominent protrusion of the nucleus on its apical surface was observed (Fig. 9(f)). Although perinuclear stress fibers appear on some cells, their numbers were highly reduced (Fig. 9(d), 9(e)).

Analyzing the above results collectively, we make a theoretical estimate of the relation between perinuclear stress fiber density and nuclear deformation.

### Nuclear Compression Model

Here we present a semi-quantitative analysis of the compression mechanism to determine how well it accounts for the observations. The estimates are made using Tatara's theory [25], [26] because of the large strain observed in confocal images. To get the estimates of compressive load we make the following simplifying assumptions

**Figure 10:**
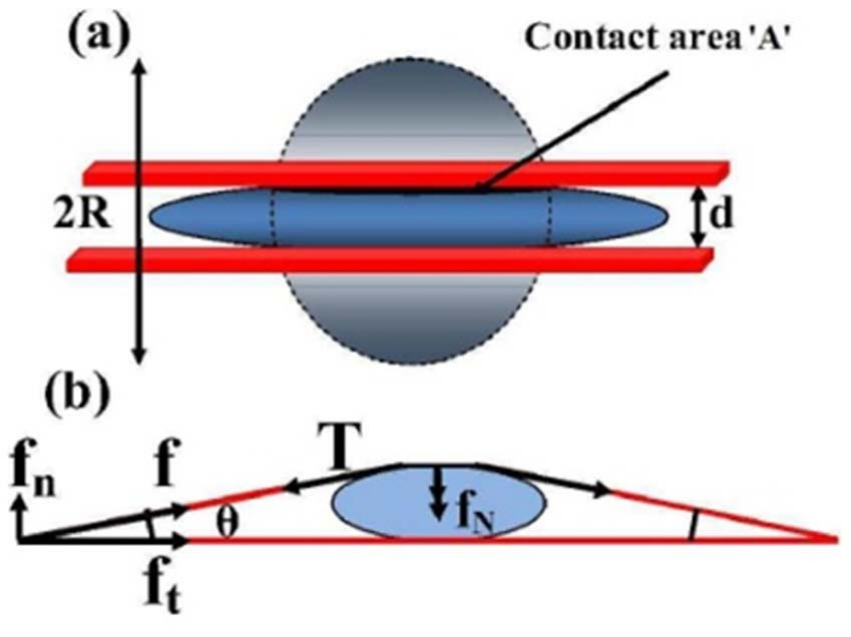
Diagrams showing the scheme used for the estimation of the compressive loading of the nucleus by stress fibers. (a) Schematic showing nuclear deformation under uniform loading. The undeformed nucleus has a radius 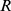. After loading the contact area with the plate is 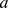 and the nuclear height is 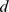. (b) Schematic showing how normal stresses arise due to a perinuclear stress fiber.

- Perinuclear stress fibers run over the nucleus in symmetric fashion and are anchored at either ends to the substrate. Tension in these fibers generate a normal stress on the nucleus. The ground state (uncompressed state) of the nucleus is taken to be a sphere (3D).
- Stress fibers running over the nucleus are assumed to be equivalent to a continuous sheet generating normal compression as shown in the schematic Fig. 10(a).
- Slipping and friction, if any, between the stress fibers and the nucleus are ignored. No slipping is supported by the fact that the stress fibers are connected to the nucleus via LINC complexes as discussed in [12].
- Nuclear volume is assumed to be constant during nuclear compression and the deformation is assumed to be elastic (viscosity is unimportant in dictating the final shape), as supported by the trypsin de-adhesion experiments. This assumption ignores effects like changes in DNA synthesis or changes in the folded structure of the nuclear membrane as discussed in [11] and [14], respectively. This assumption should be especially valid for short timescale experiments like trypsin de-adhesion, dynamic cell spreading, cytoskeletal perturbations, etc.

Below we first make an estimate of the compressive forces acting on the nucleus. The traction force per stress fiber is obtained for different substrate stiffness from the Traction Force Microscopy data (see Material and Methods and Supporting Information for details). The mean traction force 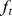 per fiber is 9.2 nN for 65 kPa, 4.4 nN for 23 kPa, and 2.8 nN for 5 kPa substrate rigidity (see S3 for distributions). This reduction in traction force with substrate stiffness is in agreement with earlier works [3], [27], [28]. We estimate the normal force due to one stress fiber as follows. From Fig. 10(b), 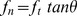, where 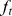 is the traction force and 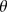 is obtained from confocal images. Therefore, the normal compression acting on the nucleus due to one stress fiber is given by 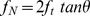. The normal force due to all the perinuclear fibers add up to give the net compressive loading on the nucleus. Stress fiber numbers vary from about 10 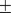 2 (n  =  15) for cells on stiff substrates to no visible fibers on the softest substrate (see Fig. 7 and Fig. S12). The net compressive load on the nucleus for stiff substrates (assuming 10 stress fibers) is calculated to be around 30 nN.

We now use Tatara analysis for a sphere compressed between two plates [29] to check whether such a loading can cause the observed deformation.
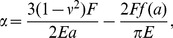
(1)where 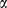  =  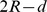 is the approach (see Fig. 10), 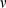  =  Poisson's ratio, a  =  contact radius, E  =  elastic modulus of the nucleus, F  =  compressive load, and
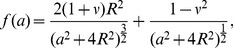
(2)where 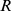, 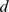 and 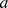 are taken from confocal images (see table S2 for values).

Using the above expressions, we obtain the Young's modulus of the nucleus to be 0.4 kPa. This is comparable to the value obtained for stem cells in recent work [30]. This means that the nuclear deformations arising from the variation in the cell spreading can be explained by the normal pressure exerted by the perinuclear stress fibers as was hypothesized in [12]. On softer substrates, both the stress fiber density and traction forces reduce as observed, resulting in reduced compressive loading and hence less nuclear deformation. A recent study using epithelial cells on adhesive islands of different aspect ratios and a 2D-model has shown that lateral stress fibers can generate forces large enough to account for geometry-dependent nuclear deformations [14]. This is analogous to the normal compression in our case.

### Nuclear Compression Experiment

**Figure 11:**
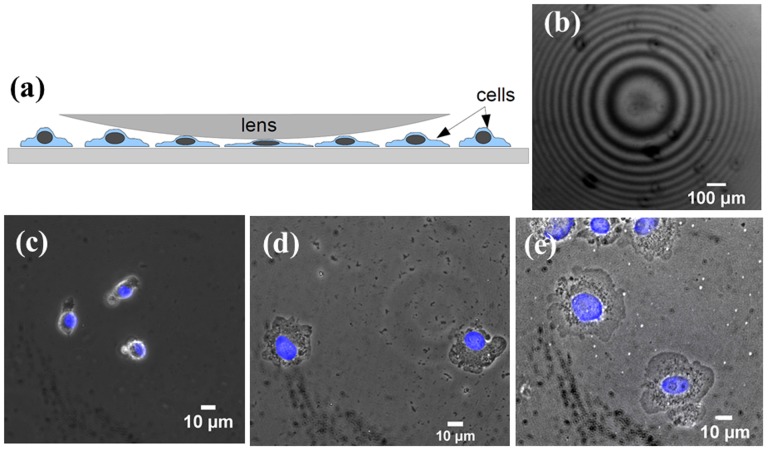
Nuclear compression experiment. (a) Schematic of the arrangement used to apply an external compressive load on the nucleus. The pressure exerted on the cells is maximum at the central point determined by observing the interference pattern in reflected light (Newton's rings) as shown in (b). A 4x objective and 200 mm focal length lens were used for this image for better illustration of the pattern. (c–e) Phase contrast images showing the effect of a compressive load on cells applied using a convex lens (c) Cells outside the lens and (d,e) cells under lenses of two different weights, 0.97 g and 1.07 g respectively. The slight reduction in image quality is due to inclusion of the lens. An increase in cell spreading and nuclear projected area was observed with increasing load, shown quantitatively in master plot Fig. 6.

Based on the above results and modeling, we hypothesize that the nucleus may be a limiting factor in cell spreading and nuclear compression may lead to enhanced cell spreading. In order to verify this we mechanically applied an external compression on the cell cortex and studied the resultant changes in nuclear deformation and cell spreading. This arrangement is shown schematically in Fig. 11(a). For this, we developed a simple technique which uses plano-convex lenses of focal length 400 mm and weights 0.97 and 1.07 grams to apply a normal pressure on the nucleus. As compared to a flat surface, a lens eliminates the need for alignment of the two surfaces (the curved surface of the lens and the coverslip with cells). For a low curvature lens (long focal length), the surface can be approximated to a locally flat one in comparison to the length scale of a cell. The lens, placed in the culture dish over a monolayer of cells (30–40% confluent) exerts a pressure proportional to its weight with a radial distribution around the bottom most point of contact. At the point of contact the surface can be assumed to be perfectly parallel to the coverslip. The point of maximum pressure is easily determined from the interference pattern (Newton's rings) generated by reflected, green, monochromatic light from the curved surface of the lens and the upper surface of the coverslip as shown in Fig. 11(b). This illumination is done using the fluorescence imaging unit of the microscope without an emission filter. The weight of the lens is chosen to apply a gentle pressure without damaging the cells. For this experiment, mMSCs were culture for 8 hrs, on coverslips coated with 0.05 *µ*g/ml of fibronectin (low concentration) to obtain an intermediate level of spreading before the application of pressure. Observations were made below the lens and outside the lens. As soon as the lens is placed on the cells, the cells began to spread (see Movie S4). In some cases the cells exhibited extensive blebbing for a short period similar to that seen in normal cell spreading (see Movie S5). The cell spreading reach a steady state within 15 min. Images of the cells under external compression are shown in Fig. 11(c–e). It is observed that a compressive loading of the cortex results in flattening of the nucleus and an increase in cell spreading. Remarkably, the data for cell and nuclear areas obtained for the two different compressive loads fall on the master-curve as shown in Fig. 6.

Below, we make an estimate of the normal compression on the cells due to a lens of mass 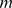. The maximum pressure due to the lens (at the central point) is given by
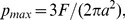
(3)where 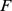 is the weight of lens after correction for buoyancy and 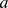 is the contact area between the lens and the layer of cells and is given by
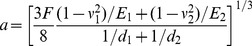
(4)as discussed in [31], [32]. Here, 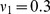 and 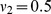 are the Poisson's ratios for the lens and the cell respectively, 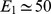 GPa and 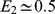 kPa are the corresponding Young's moduli, 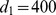 mm is the diameter of the intending object (twice the radius of curvature of the lens), and 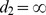, since the cell layer is flat.

F can be calculated as,
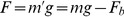
(5)

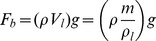
substituting in eq.5, we get
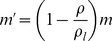




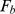
 is the buoyant force on lens; m = 1.06 g, mass of the lens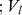 is the lens volume and 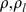 are the densities of the cell culture medium and glass respectively. This gives 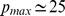 pN/*µ*m^2^. If we assume the cells to be a confluent layer and a cell-lens contact radius of 10 *µ*m per cell, the force per cell is about 7 nN. Since the cells are only 30–40% confluent the actual value could be a few times higher as the weight of the lens is supported by fewer cells. This is then comparable to the compressive force estimated earlier from traction force values which is about 3 nN per stress fiber and several of these stress fibers may be acting on the nucleus. Thus, the compressive force exerted by the lens is of the same order of magnitude as that generated by stress fibers.

## Discussion

Recent studies on fibroblasts cultured on elongated adhesive stripes of varying width has shown that perinuclear stress fibers running over the nucleus can regulate nuclear shape [12]. Further, it has been shown that endothelial cells grown on highly elongated adhesive islands exhibit lateral actin stress fibers which can generate active tension resulting in the deformation of the nucleus [14]. It is shown that the nuclear geometry depends on the aspect ratio of the adhesive island that dictate cell shape. These and similar results reveal the role of stress fibers in regulating nuclear geometry and the influence of cell shape in this [12], [14]–[16]. However, how the actin cytoskeleton or stress fibers influence cell spreading is not clear. It has been known for some time that cell spreading and nuclear geometry are related [17], [18] but the mechanism for this coupling remains poorly understood. In this article we have explored this aspect by adopting a wide range of quantitative methods.

### Cell and nuclear projected areas are robustly coupled and follow a Master Curve

Measurements of cell spreading and nuclear projected area, which is a measure of nuclear deformation, shows a robust correlation between the two. These two quantities are related over the entire measurement range. In order to test the robustness of this relationship under different spreading conditions, we measured the cell and nuclear spread (i) using cells seeded on gels of varying stiffness, (ii) by monitoring the two areas as cell spreading progressed on fibronectin coated glass surfaces, (iii) during trypsin mediated de-adhesion. All these experiments show quantitatively very similar behavior. In order to check if the two areas can be de-coupled by perturbing the cytoskeletal structure or the actin-nucleus linkages we performed a series of such experiments. Remarkably, actin or microtubule depolymerization, inhibition of active stresses by myosin-II inactivation and perturbation of nuclear–actin linkages all resulted in variations in cell spreading respecting the same relation between nuclear and cell areas as seen in normal cells. All these data follow a master-curve without the need for any scaling as shown in Fig. 6.

### Perinuclear stress fibers regulate cell spreading as well as nuclear shape

Next, we explored how nuclear deformations may be coupled to cell spreading. Imaging the actin cytoskeleton reveal that a number of perinuclear stress fibers run over the nucleus in well spread mMSCs, similar but lower in number compared to that reported for fibroblasts [12]. When cells were imaged on gels of different stiffnesses, the number of these fibers show a correlation with the extent of cell spreading, decreasing from about 10 in fully spread cells to no detectable stress fibers in the lowest range of cell spreading (softest gel). The model shows that these stress fibers can generate enough tension to deform the nucleus as seen in experiments. In poorly spread cells, stress fibers are replaced by a cortical actin layer below the membrane. These observations suggest that the compression of the nucleus by actin stress fibers may be the primary cause for the observed nuclear deformation dependent changes in cell spreading. Compression of the nucleus by applying an external load using the lens-technique demonstrates this mechanism. Indeed, the application of an external compressive loading over the nucleus resulted in increased nuclear and cell spreading. Remarkably, the data agrees well with the master-curve Fig. 6. These results reveal the importance of perinuclear stress fibers in coupling nuclear deformation to cell spreading as elaborated in the next section.

### Nuclear compression by stress fibers aid cell spreading

**Figure 12:**
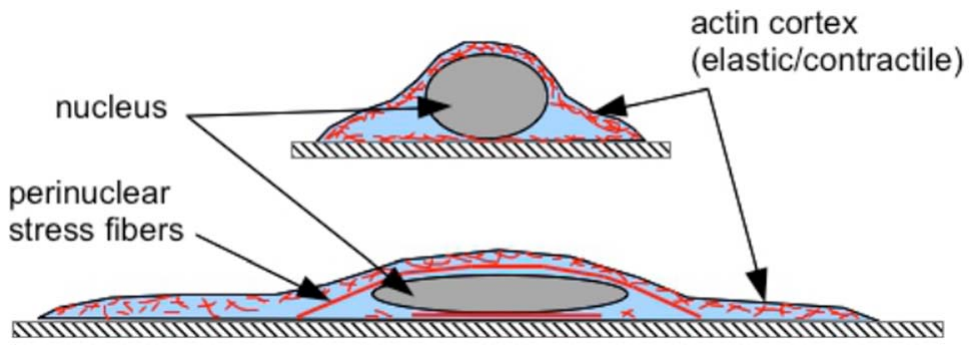
Schematic showing how nuclear compression helps in cell spreading. Flattening the nucleus by perinuclear stress fibers (also see [12]), or an external load as in the case of lens experiment, allows the cell to spread to a greater extent. In the absence of such a compressive loading of the nucleus, the nucleus exerts an upwards force on the cell cortex which constraints cell spreading due to balance of adhesive and elastic forces as elaborated in the text.

Together, our results show that a compressive loading of the nucleus by stress fibers is a mechanical regulating factor that dictates cell spreading. This can be explained as follows. A nucleus is an elastic inclusion inside a cell. In the absence of a nucleus, as in the case of cell fragments, cells are able to spread to an almost flat structure, maximizing the extent of spreading. In normal cells, the nucleus prevents the cell from reaching this maximally spread state, since deforming the nucleus costs elastic free energy. The cell achieves an intermediate state of spreading, which is dictated by the elastic deformability of the nucleus (its Young's modulus) and the substrate mediated adhesion. However, if the nucleus is compressed by an external force, the cell is able to spread to a greater extent as shown by the nuclear compression experiment using lens. In a normal cells plated on an adhesion promoting substrate, this compression is brought about by the perinuclear stress fibers as discussed in the modeling section. This is schematically shown in (Fig. 12). This simple mechanism may account for the observed correlation between cell spreading and nuclear compression by stress fibers. Similar arguments have been made previously to understand early stages of cell spreading dynamics but without considering stress fiber mediated nuclear compression [33], [34]. If the extent of actin stress fibers compressing the nucleus is reduced or if the contractility is blocked, the nucleus becomes more rounded and, thereby, reduce the ability of the cell cortex to spread out due to surface mediated forces. This agrees well with our data, the confocal observations, cell response to cytoskeletal or myosin-II perturbation experiments (Fig. 5) and nuclear compression experiment. When perinuclear stress fibers are absent, as in cells on gels, lamin knock-down cells or cells treated with low levels of latrunculin, the nucleus bulges out and exert an outward pressure on the cortex (membrane plus any cortical actin mesh) causing a reduction in spread area. In all these perturbation experiments a reduction in nuclear projected area occurs concomitant with a reduction in cell area and the data follows the same master curve (Fig. 6).

Pulling forces generated by stress fibers terminating on the nucleus may be ruled out on two accounts [17]: (i) confocal images do not show any cusp-like deformations at the attachment points which are expected if the nucleus is stretched by localized tensile stresses and (ii) these stress fibers which are attached to only one focal adhesion complex and terminate on the nucleus have a unipolar organization of actin and cannot generate contractile stresses [35], [36]. On the other hand, experiments have shown indentation of the nucleus due to perinuclear stress fibers which is expected in case of compressive stresses [16].

### Role of Myosin-II in regulating cell spreading

One of the more remarkable observations is that myosin-II inhibition produces antagonistic effects on cells plated on stiff and soft substrates (Fig. 5(b,c) and S7). Cells grown on stiff substrates decrease their spread area when myosin is inhibited whereas those plated on soft substrates show increased spreading when treated with blebbistatin. This may be understood as follows. Stress fiber numbers are high on stiff substrates and perinuclear fibers help cells spread better as already discussed and shown in Fig. 12(a). Blebbistatin treatment causes disassembly of stress fibers and therefore the compressive loading of the nucleus by perinuclear fibers (Fig. S14). This results in decreased cell spreading as discussed above. On soft substrates, on the other hand, actin is mostly in the form of a cortical mesh (as shown in Fig. 12(b)). In presence of phosphorylated myosin such a cortex is contractile and hence act as an active elastic shell [37]. This cortical tension tries to maintain a spherical shape for the cell while adhesive forces tries to increase spreading and hence cause deformation (see, for example, [38]). Inhibition of myosin-II drastically reduce cortical tension [37]. Relaxing this active tension by inhibiting myosins tips the balance towards better spreading. The mechanism by which the acto-myosin structure organizes as stress fibers on stiff substrates (well spread cells) and as cortical actin on soft substrates (poorly spread cells) require further investigation.

## Conclusion

In summary, the experiments reported here demonstrate a tight quantitative relationship between nuclear deformation and cell spreading in mMSCs. Using independent methods we showed that this relation holds irrespective of the way cell spreading is altered, under cytoskeletal and nuclear perturbations, and holds across different cell types. With the help of observations and a simple theoretical model, we hypothesize that compressive loading of the nucleus by perinuclear stress fibers can account for the increase in cell spreading with higher nuclear compression. This is demonstrated by a simple method to apply external compression to the nucleus. While these experiments help us understand how perinuclear stress fibers aid in cell spreading, understanding how the formation of stress fibers depend on substrate properties and how gene expression profile depends on nuclear geometry requires further investigations. Also, mechano-chemical coupling mediated by signalling pathways resulting in feedback processes have to be investigated to obtain a more complete picture of the role played by actin stress fibers in regulating cell and nuclear shape. However, we believe that these experiments and analysis capture the main physical mechanisms that regulate cell and nuclear deformations in a tightly coupled manner.

## Supporting Information

Figure S1
**Elastic moduli (Youngs modulus) of Polyacrylamide substrates as a function of bis-acrylamide concentration.** The calibrations have been done using AFM (1 micron dia. rounded tip) and ball-indentation method (ball dia 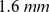) as described in the main text. The data points for the latter are averages of three spatial locations on each gel. For the AFM method, an area of 100 X 100 square micron was scanned and the data points are mean values and the error bars are standard deviations. The data values are given in Table S1.(DOCX)

Figure S2
**Image showing cells spread on a PAA substrate containing a layer of** 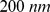 **fluorescent beads used for traction force microscopy.**
(DOCX)

Figure S3
**Histograms showing the distribution of traction stress in cells plated on substrates with three different rigidities: (a)** 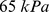 **, (b)** 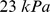 **, and (c)** 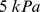 **.** Note that the traction forces become weaker with reducing substrate rigidity. “N” is the number of focal adhesions taken into account to get the traction distribution. The average focal adhesion (FA) area was found to be 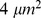 and the force per focal adhesion point was calculated by multiplying FA area to the average traction.(DOCX)

Figure S4
**(a) Displacement map obtained by tracking the fluorescent beads for the cell shown in (b).** The substrate rigidity is 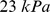. (c) Zoomed-in composite image showing bead displacements obtained before typsinization (red) and after trypsinization (green). (d) Fluorescence image of a cell transfected with Vinculin-venus taken in epifluorescence mode. Traction forces were calculated for regions containing mature focal adhesions as described in the main article.(DOCX)

Figure S5 **Comparison of cell spread area and nuclear projected area as a function of substrate stiffness for mMSC, 3T3 fibroblast and C2C12.** Each data point is an average over about 100 cells and error bars are Standard Error.
(DOCX)

Figure S6 **Alterations in nuclear shape of a cell in its adherent and non-adherent forms.** (a) & (b) show lateral and transverse view of the nucleus for a cell cultured on fibronectin coated coverslips before trypsinization. (c) & (d) show the lateral and transverse view of the same nucleus after trypsin mediated de-adhesion. Volume measurements before and after deadhesion shows volume conservation during trypsin de-adhesion as discussed in the main article.
(DOCX)

Figure S7
**Images of mMSCs taken before and after Latrunculin-A (Lat-A) treatment for 20 min.** (a) Before and (b) after treating with 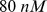 Lat-A. (c) Before and (d) after treating with 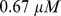 Lat-A.(DOCX)

Figure S8
**Fluorescence images (labelled with Rhodamine-phalloidin) of the actin stress fibers for control cells (a–d) and for cells treated with** 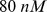 **Latrunculin-A (e–h).** At such very low concentrations, Lat-A preferentially disrupts apical stress fibers.(DOCX)

Figure S9 **Images of cells taken (a) before and (b) after treating with 0.1% DMSO.** No change in average spreading was noticed although individual cells dynamically alter their shape as a function of time as in normal medium.
(DOCX)

Figure S10
**Fluorescence images showing antagonistic changes in cell spreading upon Blebbistatin treatment for cells grown on stiff and soft substrates.** Cells treated with Blebbistatin show significantly reduced sensitivity towards the substrate stiffness and try to achieve an optimum cell spreading within one hour of Blebbistatin treatment. (a–d) and (e–h) show cells cultured on substrate with stiffness 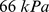 before and after Blebbistatin treatment respectively. (i–l) and (m–p) show cells cultured on a substrate with elastic modulus 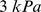 before and after Blebbistatin treatment respectively. Note that for the stiff substrate the cell spreading decreases after myosin inhibition whereas for the soft substrate it increases after treatment.(DOCX)

Figure S11 **Images showing the typical variation in stress fibers distribution or actin organization for cells grown on substrates with different rigidities.** (a–c) 65 kPa, (d–f) 23 kPa, (g–i) 5 kPa and (j–l) 3 kPa.
(DOCX)

Figure S12 **A rough estimate of the number of stress fibers can be obtained by measuring the intensity profile across the cell as shown in (a).** The line width is 5 pxl. and the image was smoothened slightly using Gaussian Blur of 2 pxl. size using ImageJ to reduce noise. The line profile thus obtained is shown in (b).
(DOCX)

Figure S13
**Effect of TSA on mMSCs.** (a) and (b) are the composite images of a cell and the corresponding nucleus before and after 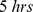 of TSA treatment.(DOCX)

Figure S14 **Images showing that stress fibers are absent in cells treated with blebbistatin irrespective of substrate rigidity.**
(DOCX)

Movie S1
**Change in cell area and nuclear area as a function of time during cell spreading.** Cells were cultured on coverslips coated with 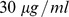 fibronectin solution for 1 hr and incubated at 37°C. The total duration of the clip is 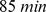.(AVI)

Movie S2
**A** 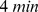 **(actual duration) video showing the detachment of a cell by trypsin treatment.** Cells were culture on fibronectin coated cover slips for 24 hrs before the experiment. Experiment was carried out at 37°C.(AVI)

Movie S3
**Time lapse video taken under Lat-A treatment.** Lat-A concentration used was 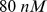 and the total duration of the clip is 30 min. The cell stops retracting and the spreading reaches saturation after 20 minutes.(AVI)

Movie S4
**Evolution of cell spreading under an imposed compressive load applied using a lens of weight** 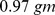 **, as a function of time.** Note that the region of maximum compression can be determined only after placing the lens and hence cell spreading has proceeded significantly by the time the recording was started. The quantification of spreading in the Master plot Fig. 6 is done for cells outside the lens and those below the lens after saturation in spreading. The total duration is 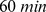. The floating particles are the debris from the previous trials of loading the cells with lens.(AVI)

Movie S5
**Evolution of cell spreading under an imposed compressive load applied using a lens of weight** 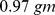 **, as a function of time.** Note that the region of maximum compression can be determined only after placing the lens and hence cell spreading has proceeded significantly by the time the recording was started. In this case extensive blebbing can be seen during the initial stages. Duration of the movie is 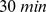.(AVI)

Table S1 **Comparison between the two methods of determining youngs modulus of PAA gel. See Fig. S1 and description in main text for details.**
(DOCX)

Table S2 **Typical values of the parameters used in the model (** **Eqn. 1** **and** **2** **).** Method of calculating the traction and the normal force have been discussed in Materials and Methods and Supporting Information. Values are measured from the confocal images.
(DOCX)
