# Correction: Role of Actin Filaments in Correlating Nuclear Shape and Cell Spreading

**DOI:** 10.1371/journal.pone.0119076

**Published:** 2015-03-23

**Authors:** The PLOS ONE Staff

In Fig. 1(a), one layer of the plot is incorrectly shifted slightly on the x-axis. The authors have provided a corrected version here.

**Fig 1:**
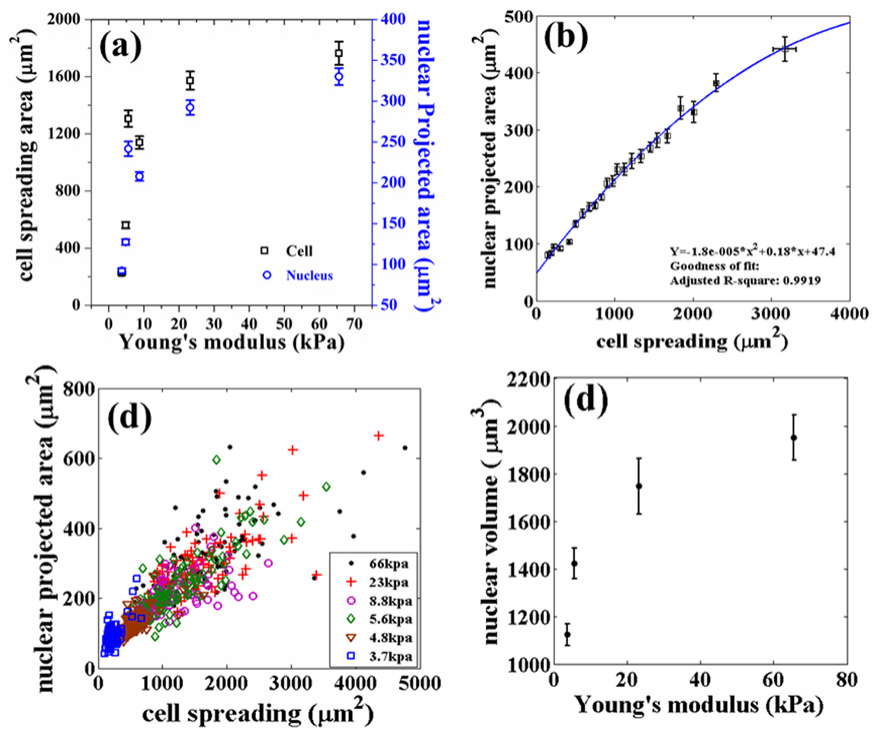
Variation of cell spreading, nuclear projected area and nuclear volume studied using gels of different stiffnesses. (a) Cell and nuclear projected area as a function of Young’s modulus of the substrate. Each point is an average taken over 100 cells. (b) Dependence of nuclear projected area on cell spreading obtained after putting all the data from all rigidities together and then binning the data points for cell spreading. Note, the difference in maximum spread area between the two figures arises due to this pooling and binning of data according to cell spread area. Binning size used was 26 and *R* ^2^ is calculated using the curve fitting toolbox, MATLAB. (c) Scatter plot (raw data) of the two areas of individual cells obtained from different substrates (same data as in a and b). Note that the range of measured cell area increases with substrate stiffness. (d) Nuclear volume as a function of the elastic modulus of the substrate measured from confocal stacks as describes in the text (20 cells for each data point). Error bars in all the plots represent mean ± standard error (SE).
