# Supplementary figures and images for: Role of Actin Filaments in Correlating Nuclear Shape and Cell Spreading
Source: PLoS One. 2014 Sep 24;9(9):e107895. doi: 10.1371/journal.pone.0107895 (PMC4177564; doi:10.1371/journal.pone.0107895)

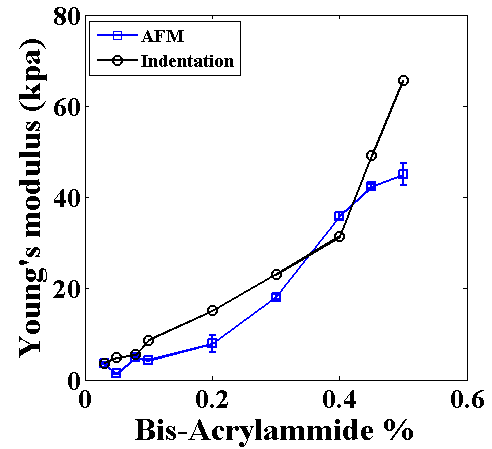

Supplement: Figure S1 — Elastic moduli (Youngs modulus) of Polyacrylamide substrates as a function of bis-acrylamide concentration. The calibrations have been done using AFM (1 micron dia. rounded tip) and ball-indentation method (ball dia ) as described in the main text. The data points for the latter are averages of three spatial locations on each gel. For the AFM method, an area of 100 X 100 square micron was scanned and the data points are mean values and the error bars are standard deviations. The data values are given in Table S1. (DOCX) [file pone.0107895.s001.docx]

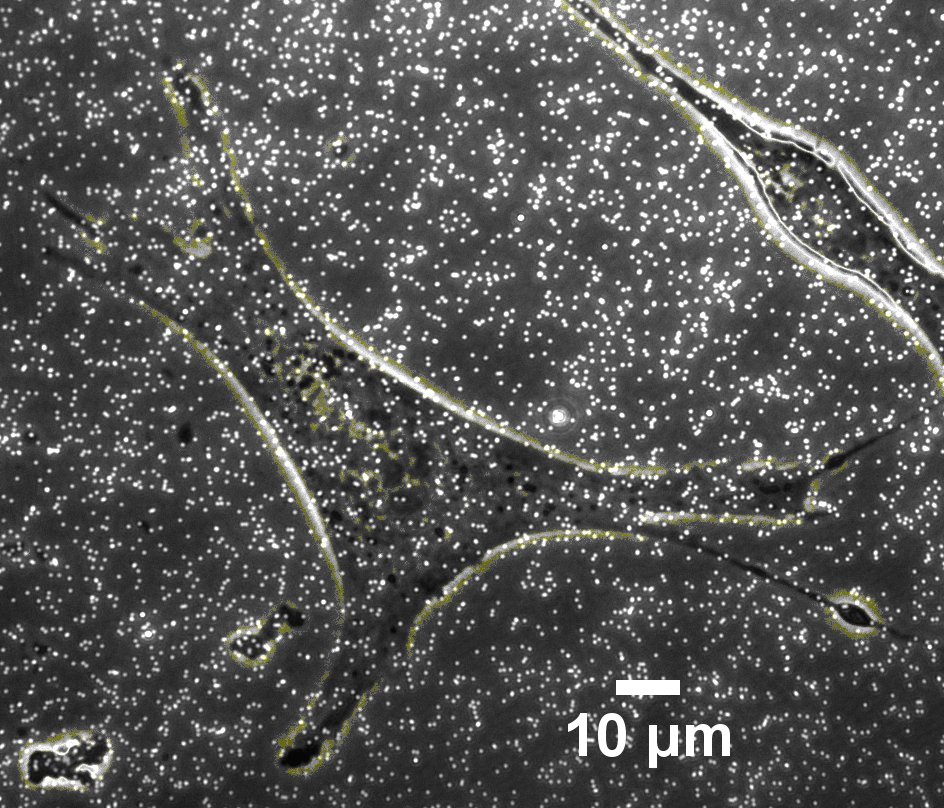

Supplement: Figure S2 — Image showing cells spread on a PAA substrate containing a layer of fluorescent beads used for traction force microscopy. (DOCX) [file pone.0107895.s002.docx]

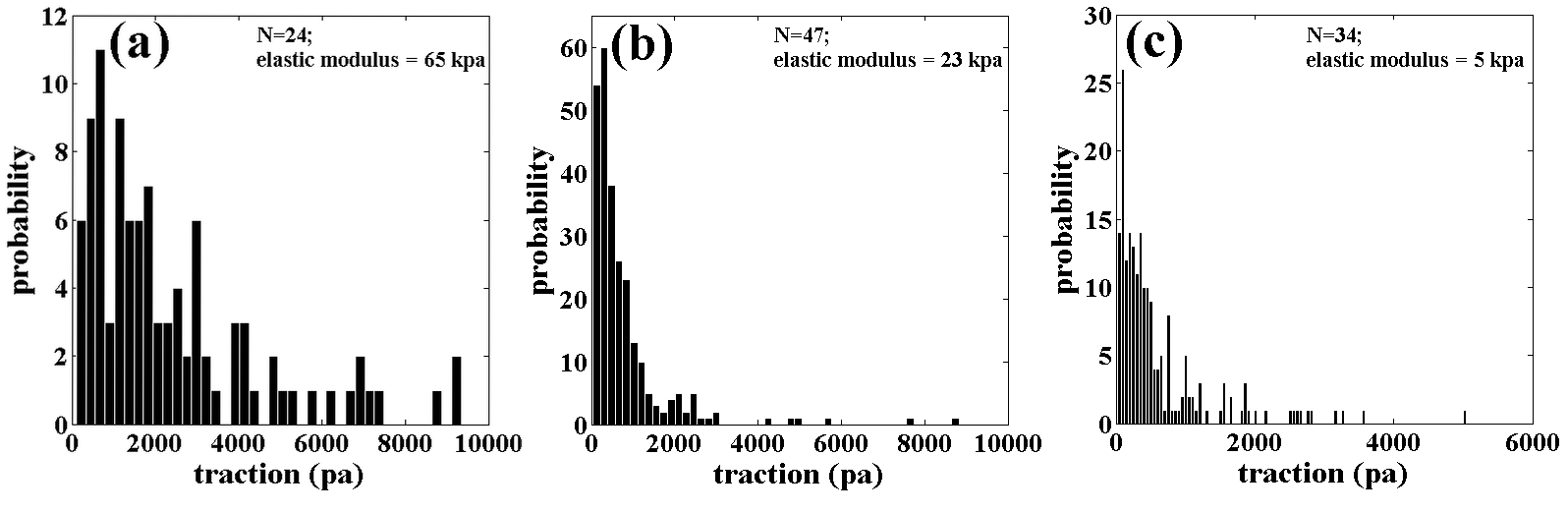

Supplement: Figure S3 — Histograms showing the distribution of traction stress in cells plated on substrates with three different rigidities: (a) , (b) , and (c) . Note that the traction forces become weaker with reducing substrate rigidity. “N” is the number of focal adhesions taken into account to get the traction distribution. The average focal adhesion (FA) area was found to be and the force per focal adhesion point was calculated by multiplying FA area to the average traction. (DOCX) [file pone.0107895.s003.docx]

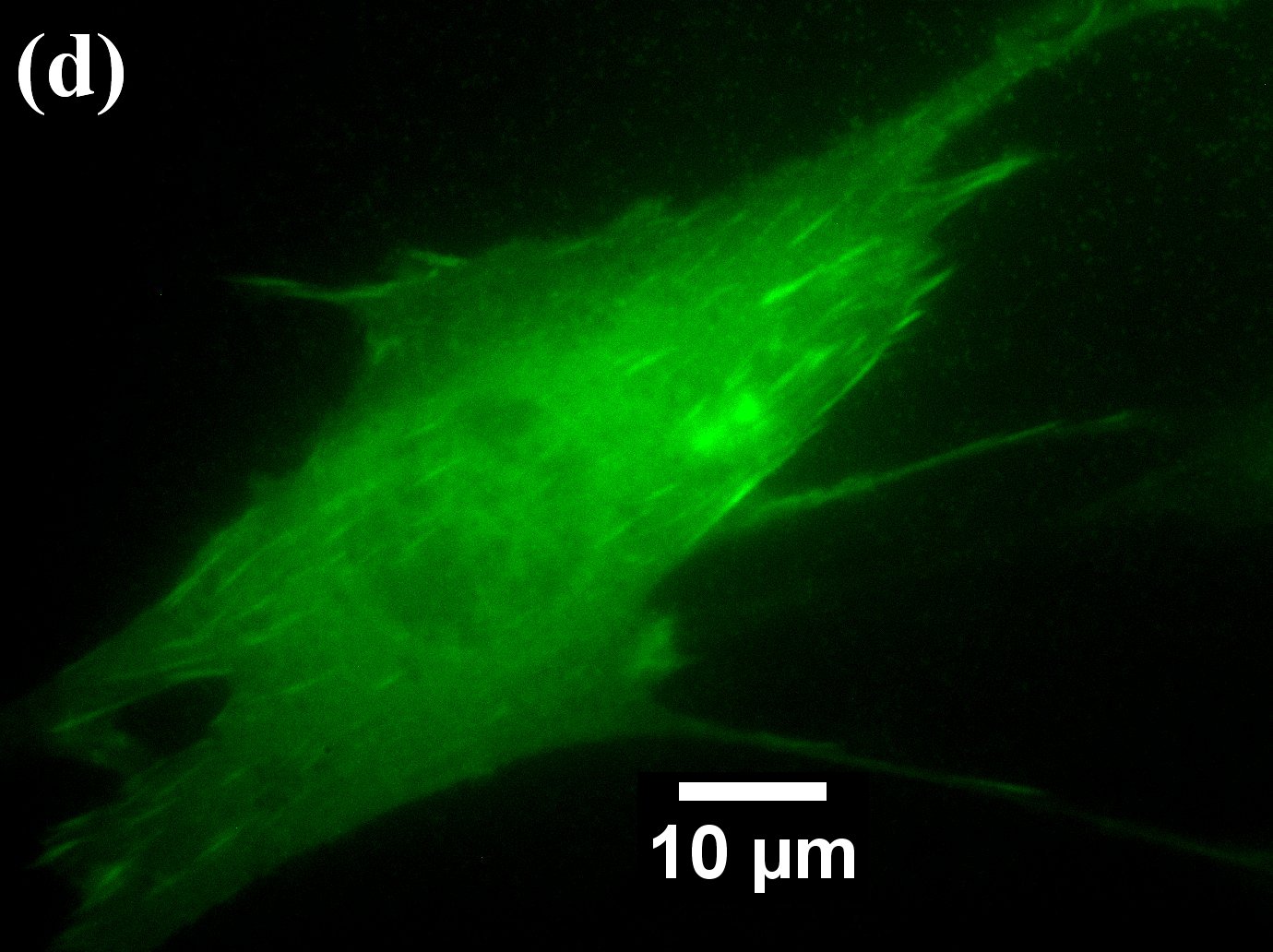

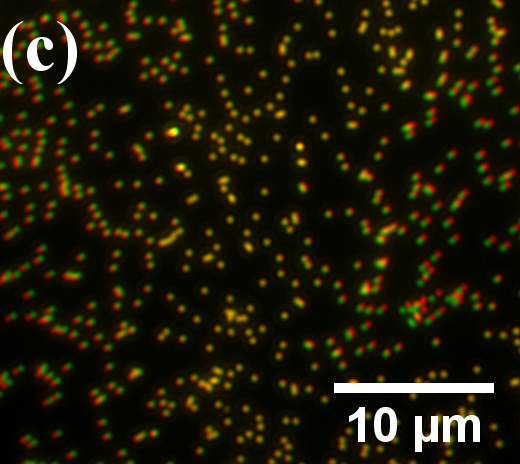

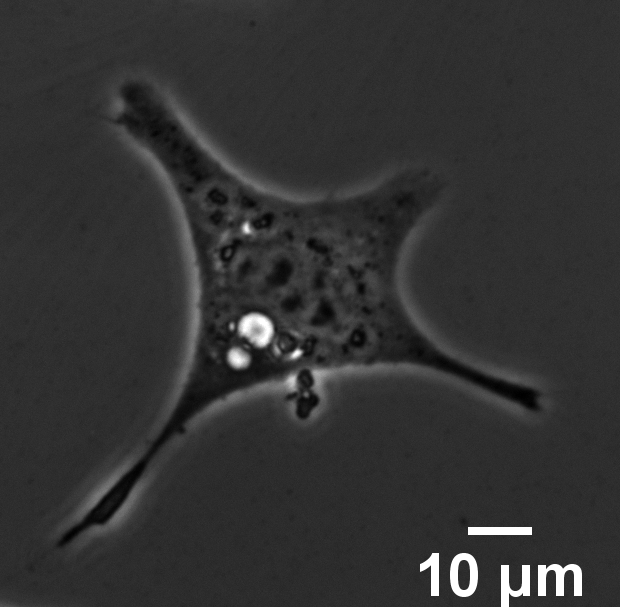

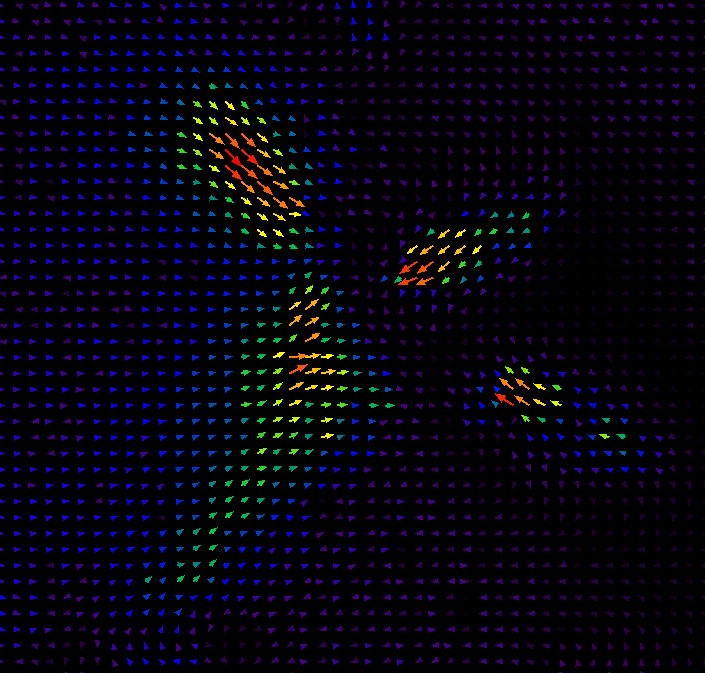

Supplement: Figure S4 — (a) Displacement map obtained by tracking the fluorescent beads for the cell shown in (b). The substrate rigidity is . (c) Zoomed-in composite image showing bead displacements obtained before typsinization (red) and after trypsinization (green). (d) Fluorescence image of a cell transfected with Vinculin-venus taken in epifluorescence mode. Traction forces were calculated for regions containing mature focal adhesions as described in the main article. (DOCX) [file pone.0107895.s004.docx]

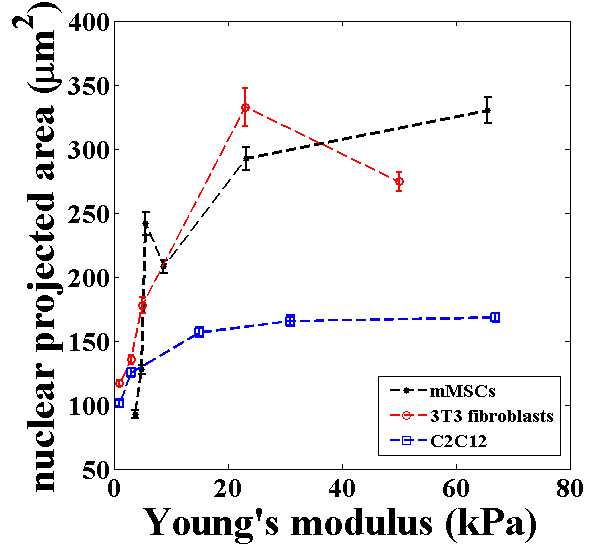

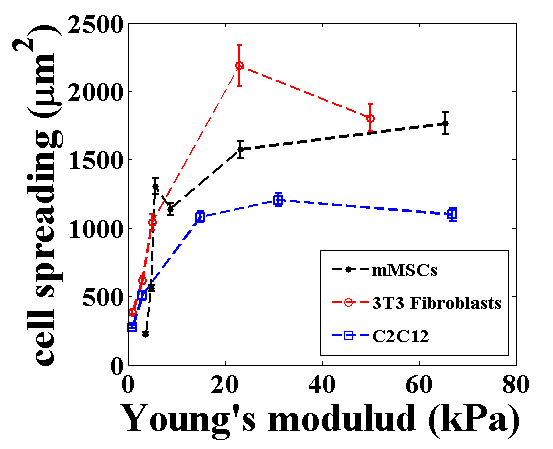

Supplement: Figure S5 — Comparison of cell spread area and nuclear projected area as a function of substrate stiffness for mMSC, 3T3 fibroblast and C2C12. Each data point is an average over about 100 cells and error bars are Standard Error. (DOCX) [file pone.0107895.s005.docx]

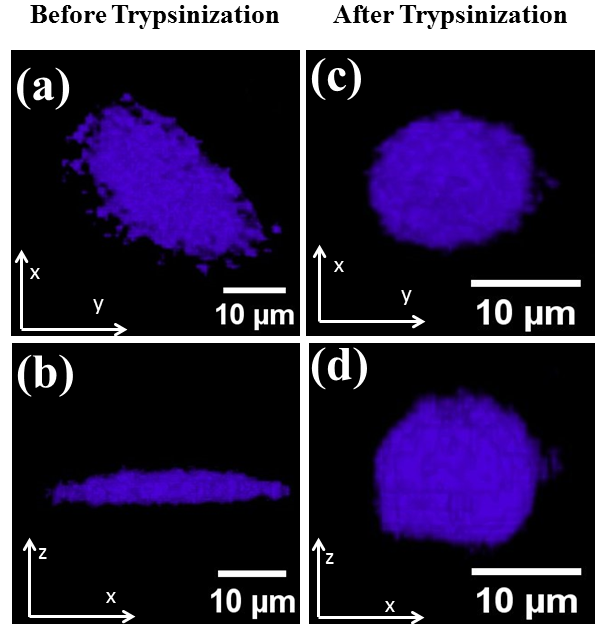

Supplement: Figure S6 — Alterations in nuclear shape of a cell in its adherent and non-adherent forms. (a) & (b) show lateral and transverse view of the nucleus for a cell cultured on fibronectin coated coverslips before trypsinization. (c) & (d) show the lateral and transverse view of the same nucleus after trypsin mediated de-adhesion. Volume measurements before and after deadhesion shows volume conservation during trypsin de-adhesion as discussed in the main article. (DOCX) [file pone.0107895.s006.docx]

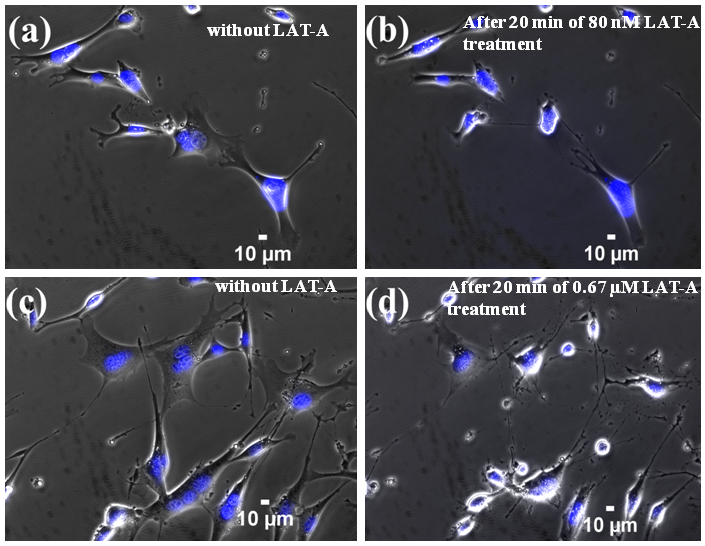

Supplement: Figure S7 — Images of mMSCs taken before and after Latrunculin-A (Lat-A) treatment for 20 min. (a) Before and (b) after treating with Lat-A. (c) Before and (d) after treating with Lat-A. (DOCX) [file pone.0107895.s007.docx]

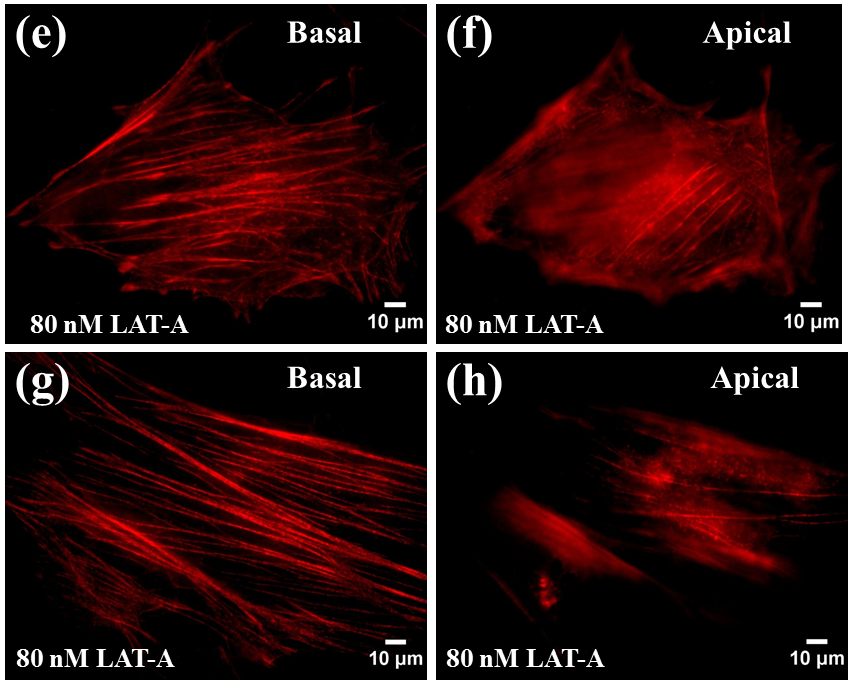

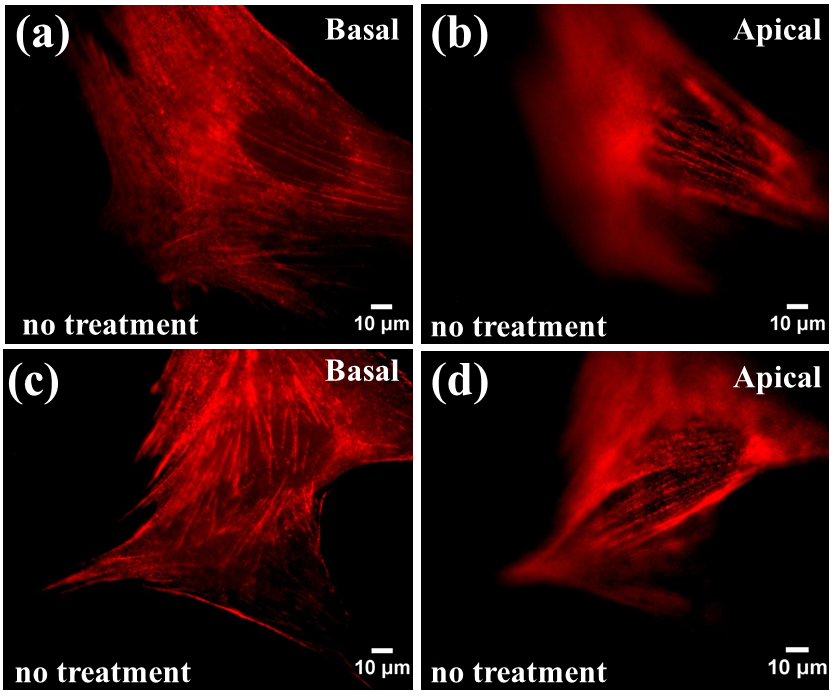

Supplement: Figure S8 — Fluorescence images (labelled with Rhodamine-phalloidin) of the actin stress fibers for control cells (a–d) and for cells treated with Latrunculin-A (e–h). At such very low concentrations, Lat-A preferentially disrupts apical stress fibers. (DOCX) [file pone.0107895.s008.docx]

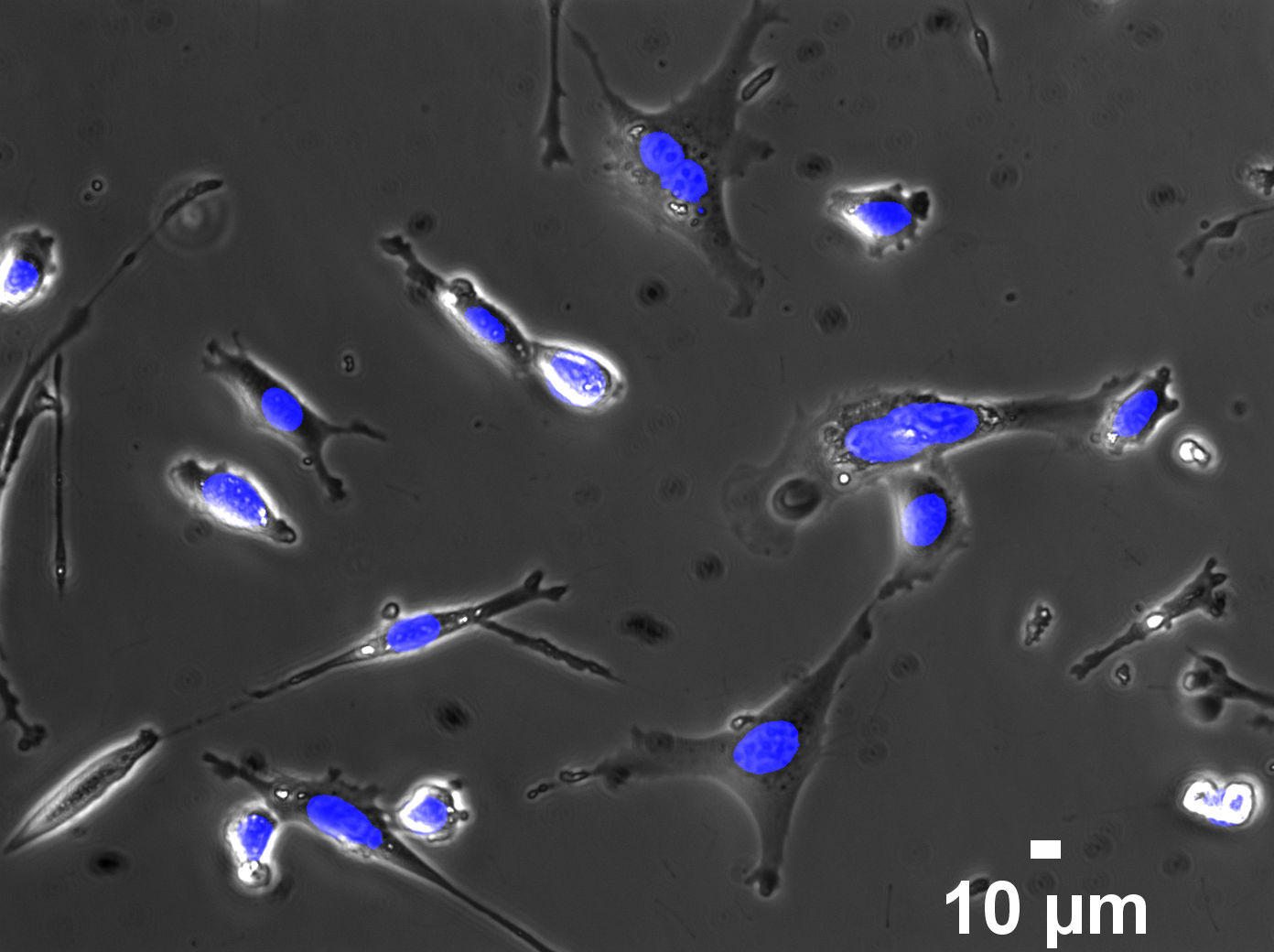

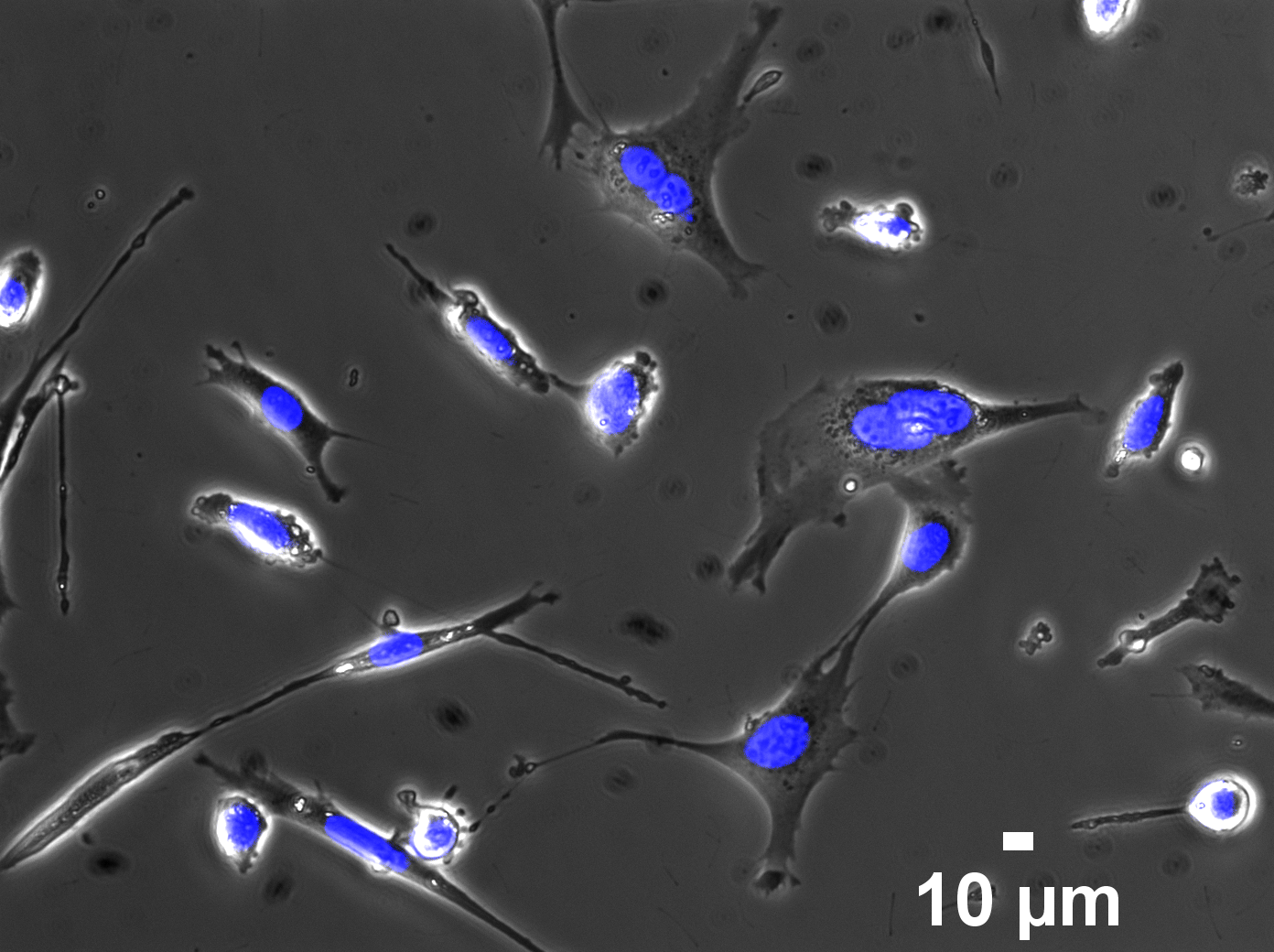

Supplement: Figure S9 — Images of cells taken (a) before and (b) after treating with 0.1% DMSO. No change in average spreading was noticed although individual cells dynamically alter their shape as a function of time as in normal medium. (DOCX) [file pone.0107895.s009.docx]

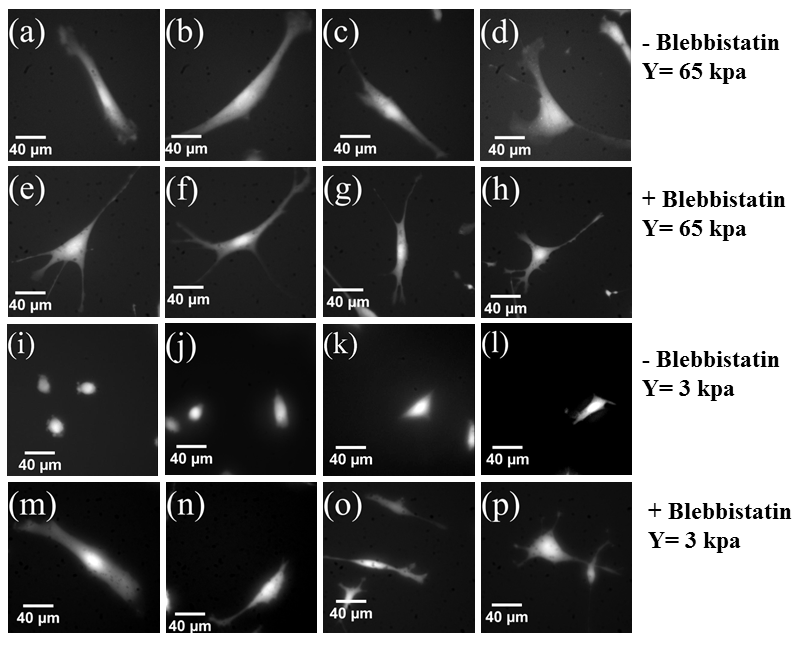

Supplement: Figure S10 — Fluorescence images showing antagonistic changes in cell spreading upon Blebbistatin treatment for cells grown on stiff and soft substrates. Cells treated with Blebbistatin show significantly reduced sensitivity towards the substrate stiffness and try to achieve an optimum cell spreading within one hour of Blebbistatin treatment. (a–d) and (e–h) show cells cultured on substrate with stiffness before and after Blebbistatin treatment respectively. (i–l) and (m–p) show cells cultured on a substrate with elastic modulus before and after Blebbistatin treatment respectively. Note that for the stiff substrate the cell spreading decreases after myosin inhibition whereas for the soft substrate it increases after treatment. (DOCX) [file pone.0107895.s010.docx]

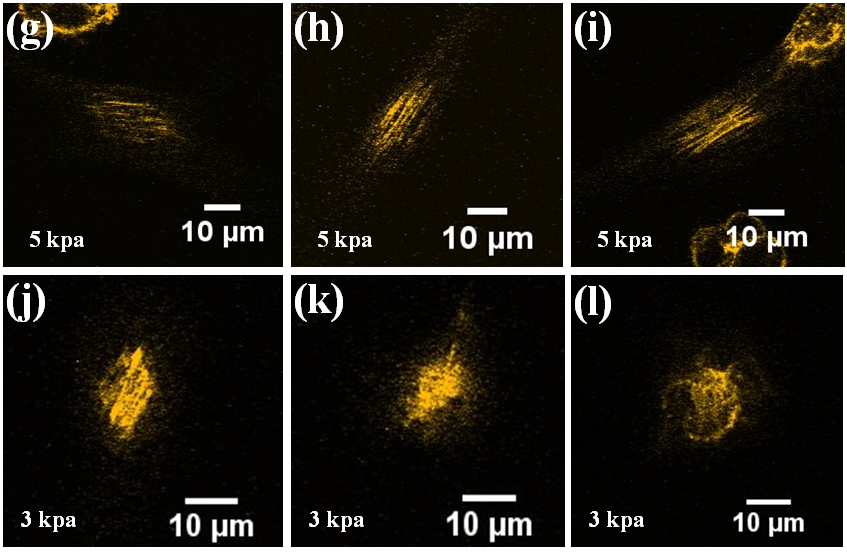

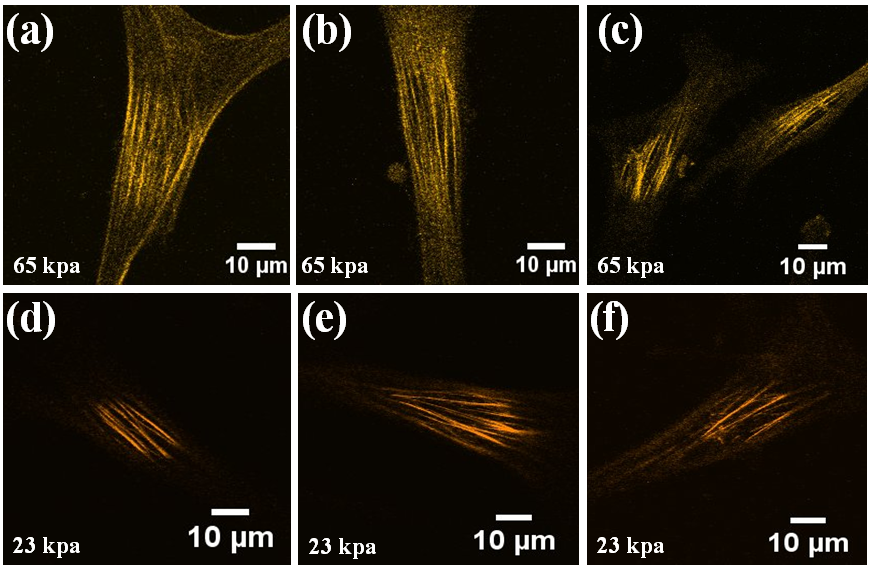

Supplement: Figure S11 — Images showing the typical variation in stress fibers distribution or actin organization for cells grown on substrates with different rigidities. (a–c) 65 kPa, (d–f) 23 kPa, (g–i) 5 kPa and (j–l) 3 kPa. (DOCX) [file pone.0107895.s011.docx]

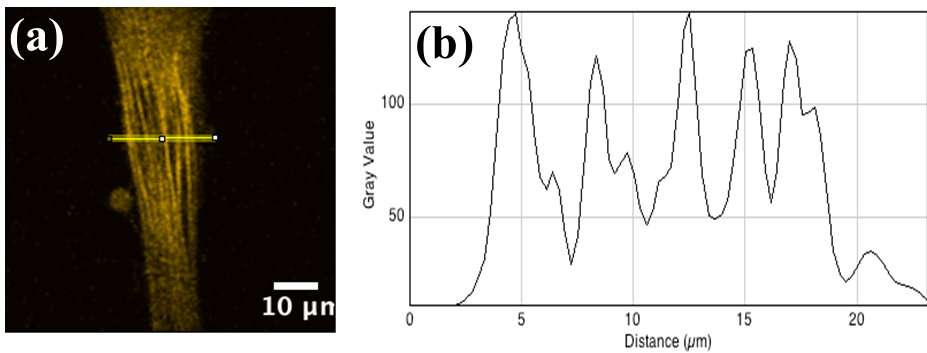

Supplement: Figure S12 — A rough estimate of the number of stress fibers can be obtained by measuring the intensity profile across the cell as shown in (a). The line width is 5 pxl. and the image was smoothened slightly using Gaussian Blur of 2 pxl. size using ImageJ to reduce noise. The line profile thus obtained is shown in (b). (DOCX) [file pone.0107895.s012.docx]

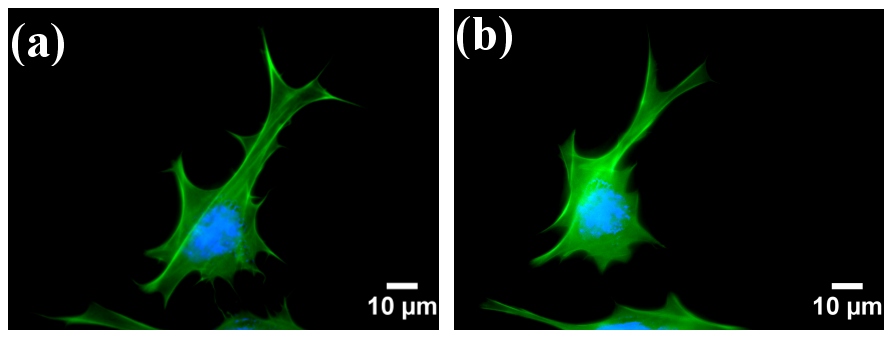

Supplement: Figure S13 — Effect of TSA on mMSCs. (a) and (b) are the composite images of a cell and the corresponding nucleus before and after of TSA treatment. (DOCX) [file pone.0107895.s013.docx]

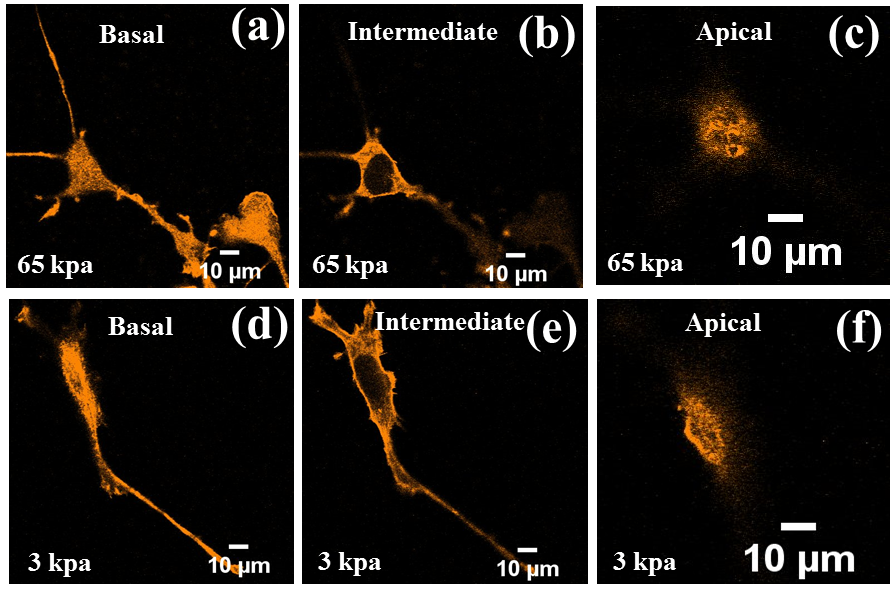

Supplement: Figure S14 — Images showing that stress fibers are absent in cells treated with blebbistatin irrespective of substrate rigidity. (DOCX) [file pone.0107895.s014.docx]
