# Supplementary material for: Role of Actin Filaments in Correlating Nuclear Shape and Cell Spreading
Source: PLoS One. 2014 Sep 24;9(9):e107895. doi: 10.1371/journal.pone.0107895 (PMC4177564; doi:10.1371/journal.pone.0107895)
Supplement: Table S1 — Comparison between the two methods of determining youngs modulus of PAA gel. See Fig. S1 and description in main text for details. (DOCX) [file pone.0107895.s020.docx]

| S.No. | Acryl%/ bis% | Young’s modulii (kpa)  AFM/Indentation |
| --- | --- | --- |
| 1. | 10/0.5 | 44.99/65.56 |
| 2. | 10/0.45 | 42.41/49.18 |
| 3. | 10/0.4 | 35.93/31.47 |
| 4. | 10/0.3 | 18.18/23.18 |
| 5. | 10/0.2 | 7.94/14.5 |
| 6. | 10/0.1 | 4.33/8.76 |
| 7. | 10/0.08 | 4.93/5.57 |
| 8. | 10/0.05 | 1.31/4.83 |
| 9. | 10/0.03 | 3.59/3.71 |
