# Supplementary material for: Role of Actin Filaments in Correlating Nuclear Shape and Cell Spreading
Source: PLoS One. 2014 Sep 24;9(9):e107895. doi: 10.1371/journal.pone.0107895 (PMC4177564; doi:10.1371/journal.pone.0107895)
Supplement: Table S2 — Typical values of the parameters used in the model ( Eqn. 1 and 2 ). Method of calculating the traction and the normal force have been discussed in Materials and Methods and Supporting Information. Values are measured from the confocal images. (DOCX) [file pone.0107895.s021.docx]

| Parameter | Value (for 65 kPa) | Error (n = 15 cells) |
| --- | --- | --- |
| Traction (kpa) | 2.3 | ±215 SE (see distribution above) |
| Radius of nucleus without compression (µm) | 8.5 | ±1.6 Stand. Dev. |
| Contact radius (µm) | 10.1 | ±1.1 Stand. Dev. |
| Nucler height (µm) | 10.8 | ±1.2 Stand. Dev. |
| Approach = 2R-nuclear height (µm) | 6.2 | ±2.3 Stand. Dev. |
